# Impact of Polymer Physicochemical Features on the Amorphization and Crystallization of Citric Acid in Solid Dispersions

**DOI:** 10.3390/polym17030310

**Published:** 2025-01-24

**Authors:** Seda Arioglu-Tuncil, Lisa J. Mauer

**Affiliations:** 1 Department of Nutrition and Dietetics, Necmettin Erbakan University, Meram, Konya 42090, Türkiye; seda.tuncil@erbakan.edu.tr; 2 Department of Food Science, Purdue University, West Lafayette, IN 47907, USA

**Keywords:** polymer, amorphous solid dispersion, carboxymethylcellulose, pectin, crystallization inhibition, intermolecular interaction

## Abstract

The amorphization and crystallization of citric acid in the presence of a variety of polymers were investigated. Polymers were chosen for their different physicochemical features, including hygroscopicity, glass transition temperature (T_g_), and functional groups capable of forming intermolecular non-covalent interactions with citric acid. Citric acid solutions with varying amounts of pectin (PEC), guar gum (GG), κ-carrageenan (KG), gelatin (GEL), (hydroxypropyl)methylcellulose (HPMC), and carboxymethylcellulose sodium (CMC-Na) were lyophilized. Dispersions were stored for up to 6 months in controlled temperature and relative humidity environments and periodically monitored using powder X-ray diffraction, differential scanning calorimetry, and Fourier transform infrared spectroscopy. Moisture sorption isotherms and moisture contents were determined. Amorphous solid dispersions of citric acid were successfully formed in the presence of ≥20% *w*/*w* CMC-Na and PEC or ≥30% *w*/*w* of the other polymers except KG which required a minimum of 40% polymer. All samples remained amorphous even in their rubbery state at 0% RH (25 °C and 40 °C), but increasing the RH to 32% RH resulted in citric acid crystallization in the KG dispersions, and further increasing to 54% RH resulted in crystallization in all samples. Polymer effectiveness for inhibiting citric acid crystallization was CMC-Na > PEC ≥ GEL > HPMC > GG > KG. To create and maintain amorphous citric acid, polymer traits in order of effectiveness were as follows: greater propensity for intermolecular non-covalent interactions (both ionic and hydrogen bonding) with the citric acid, carbonyl groups, higher T_g_, and then lower hygroscopicity.

## 1. Introduction

Citric acid is an organic acid which is naturally present in a variety of fruits and vegetables and is distributed as a crystalline ingredient in anhydrous and monohydrate forms. Citric acid has GRAS (generally recognized as safe) status and is used as a flavor enhancer, acidulant, chelating agent, and antioxidant in various products in the food and pharmaceutical industries [1,2]. Citric acid has been widely studied as a plasticizer, as a compatibilizer, and as a cross-linker in polymeric films to develop biodegradable and sustainable packaging materials [3,4,5,6], as well as to improve the microhardness of composite materials used in medical applications [7]. It has also been used as an extracting agent in plant-derived cosmetic formulations [8], and as a pH modulator and cross-linking agent in pharmaceutical products [9,10,11,12].

When food, pharmaceutical, polymeric film, and other products are processed, and particularly with the addition and removal of water and/or heat, such as in freeze drying and spray drying or film casting [13], initially crystalline ingredients can be solidified in the amorphous state, as can compounds naturally found in fruits and vegetables. Different physical forms of an ingredient (anhydrate crystal, hydrate crystal, amorphous form) exhibit different properties, including temperature sensitivity, solubilities, stabilities, dissolution kinetics [13,14,15], and sensory perceptions [16]. Crystalline solids exhibit a characteristic first-order phase transition at their melting temperature, whereas amorphous solids are softened at a temperature lower than the melting temperature, known as the ‘glass transition temperature’ (T_g_). Amorphous forms often have faster dissolution rates and higher solubilities, as well as a higher hygrocapacity at low and intermediate relative humidities (RHs), than their crystalline counterparts [17,18,19]. This is attributed to the lack of long range atomic order and crystal lattice energy, as well as the higher free volume found in amorphous structures [20].

When an ingredient can adopt different physical states, phase transformations can become problematic for product quality and shelf-life. An RH-temperature phase diagram was generated for crystalline citric acid to document the environmental boundaries at which citric acid crystals will hydrate, dehydrate, or deliquesce [21]. Regarding amorphous citric acid, studies have investigated the glass forming ability of citric acid in solid dispersions prepared by solvent evaporation and melt-quenching methods [22,23,24,25,26,27,28,29]. Amorphous solids have a tendency to crystallize over time [30]. Citric acid has a T_g_ below ambient temperature (11 °C) and a high crystallization tendency on its own [25]. However, the presence of various polymers in solid dispersions has been shown to delay crystallization of small molecules [31]. There are a few studies in which citric acid was used as a carrier and the physical stability of its amorphous dispersions was monitored over time. For example, the solid dispersions of citric acid with loratadine and paracetamol were reported to remain amorphous for three months at 0% and 60% RH at 25 °C, and 27 weeks in dry conditions, respectively [24,32]. Amorphous stability of the dispersions was attributed to the intermolecular hydrogen bonding [24,32] and elevated T_g_ [32].

The potential for citric acid to solidify in the amorphous state has implications to the widespread number of products it is added to, including food, pharmaceutical, and cosmetic products that contain a variety of ingredients, including polymers, as well as biobased polymeric films. Therefore, it is of interest to explore citric acid in solid dispersions with polymers with different physicochemical features (hygroscopicity, hydrogen bonding ability, and T_g_) to determine if the trends in polymer crystallization inhibition for different classes of small molecules (including thiamine chloride hydrochloride, ascorbic acid, resveratrol, and curcumin) [33,34,35,36] hold true for citric acid.

## 2. Materials and Methods

### 2.1. Materials

Citric acid anhydrous (catalog number: CX1723) and κ-carrageenan (KG—catalog number: C1804) were obtained from VWR Scientific (Radnor, PA, USA). Guar gum (GG—catalog number: G4129), (hydroxypropyl) methyl cellulose (HPMC—catalog number: 423238), carboxymethylcellulose sodium (CMC-Na—catalog number: C5678), and pectin (PEC—catalog number: P9135) (from citrus peel with a ~61% degree of esterification) were purchased from Sigma-Aldrich Inc. (St. Louis, MO, USA). This pectin was in the form of poly-D-galacturonic acid methyl ester. The galacturonic acid content was at least 74% (dry basis). Gelatin (GEL—catalog number: 470301-132) was obtained from Ward’s Science (Rochester, NY, USA). Drierite^TM^, used to create 0% RH storage conditions, was obtained from W.A. Hammond Drierite Company, LTD (Xenia, OH, USA). Magnesium chloride (MgCl_2_, 32% RH at 25 °C) and magnesium nitrate (Mg (NO_3_)_2_, 54% RH at 25 °C), that were used to create specific RH conditions in desiccators, were purchased from Sigma-Aldrich Inc. (St. Louis, MO, USA). All chemicals used were analytical grade.

### 2.2. Formation of Solid Dispersions via Lyophilization

Citric acid solid dispersions in polymers were initially prepared using a 1:1 weight ratio of anhydrous citric acid and the polymer. Polymers (0.25 g) were weighted and added to 30 mL of deionized water. Then, heat was applied using a digital heat block (VWR International LLC., Bristol, CT, USA) at 60 °C for 30 min, to solubilize the polymers properly. The solutions were cooled to the room temperature, and 0.25 g of citric acid was then added. Citric acid–polymer solutions were then mixed with a Roto-Shake Genie^®^ SI-1100 (Scientific Industries, Inc., Bohemia, NY, USA) until uniform one-phase solutions were visually confirmed. Further studies used samples containing different ratios of citric acid and polymer, from 0% to 100%, in 10% increments, keeping the total solids at 0.5 g added to 30 mL water. For the controls, lyophilized citric acid and polymers were prepared by dissolving 0.5 g of the individual ingredients in 30 mL of water. Then, the solutions were kept at −20 °C for at least 40 h prior to lyophilization. The lyophilization technique was conducted using a VirTis BenchTop K (VirTis, Gardiner, NY, USA) for at least 96 h at ambient temperature and 20 mT. After the lyophilization process completed, solid dispersions were transferred into 20 mL glass vials and placed into desiccators with select %RH conditions. In addition, physical blends of crystalline citric acid with the polymers were prepared by weighing each ingredient separately, followed by simply mixing in 20 mL glass vials.

### 2.3. Storage Treatments

Solid dispersions were stored in select %RH conditions (0%, 32%, and 54% RHs) at two temperatures (25 °C and 40 °C) for stability studies. A water jacketed incubator (Forma Scientific, Inc., Marietta, OH, USA) was used to maintain the 40 °C. The physical stability of the amorphous solid dispersions was analyzed weekly for up to 189 days.

### 2.4. Powder X-Ray Diffraction (PXRD)

Solid state characterization of the samples was performed immediately after lyophilization and at set time points during the storage study with a Rigaku Smartlab^TM^ diffractometer (Rigaku Americas, Woodlands, TX, USA) Cu-Kα radiation source and D/teX ultra-detector. Samples were scanned between 5–40° 2θ at 4°/min with a 0.02° step size. Solid dispersions with a diffuse halo in the diffractograms were characterized as XRD amorphous, and samples containing sharp peaks at least two standard deviations above baseline were labeled as crystalline.

### 2.5. Fourier Transform Infrared Spectroscopy (FTIR)

The interactions between citric acid and polymers were investigated using a ThermoNicolet Nexus 670 FT-IR spectrometer (Thermo Fisher Scientific, Madison, WI, USA) equipped with an MCTA detector and DRIFTS Avatar Diffuse Reflectance Smart Accessory (Thermo Fisher Scientific, Madison, WI, USA). An IR spectrum of KBr was collected before each series of analyses as a background. The scan range was set from 4000 to 650 cm^−1^, and 128 scans were recorded with a resolution of 2 cm^−1^. Prior to placement in the DRIFTS accessory, polymers, citric acid, freeze dried citric acid, citric acid–polymer dispersions, and citric acid–polymer physical mixtures were weighted (5% *w*/*w* of KBr) and then samples were milled for 30 s in a screw type capsule with a stainless-steel ball pestle, using a Crescent Digital Wig-L-Bug C020200 Mixer (Dentsply Rinn Inc., Elgin, IL, USA). The spectra were then analyzed using OMNIC software 6.1. (Thermo Fisher Scientific, Madison, WI, USA).

### 2.6. Moisture Sorption Isotherm Analysis

Moisture sorption isotherms were collected using an SPSx-1µ Dynamic Vapor Sorption Analyzer (Projekt Messtechnik, Ulm, Germany), with the equilibrium criteria set at a weight change of 0.01% in 15 min and maximum step time of 5 h. Duplicate samples (100–300 mg) were loaded into the aluminum pans and were first equilibrated at 5% RH in the instrument prior to the analysis. Samples were then analyzed from 5–95% RH and 5–80% RH at 25 °C and 40 °C, respectively, using a step size of 5% RH. An additive model, representing the weighted average moisture sorption of each individual ingredient, was used to generate predicted moisture sorption isotherm behaviors, which were compared to the experimental moisture sorption isotherms of solid dispersions. The differences between the moisture sorption of the polymers and the solid dispersions (φ) were also determined as follows:

(1) $$\varphi =m_{dispersion}-cm_{polymer}$$

where m is the % moisture content (*w*/*w*), and c is the fraction of the polymer in the solid dispersion. This approach has been used when the moisture sorption profile of the compound of interest cannot be collected from the amorphous form alone due to its tendency to crystallize [34,37].

### 2.7. Differential Scanning Calorimetry (DSC)

Glass transition temperatures (T_g_) of the samples in hermetically sealed pans were measured using a Discovery DSC (TA Instruments, New Castle, DE, USA) equipped with a refrigerated cooling accessory. Nitrogen at 50 mL/min served as the purge gas. A sample size of 7–12 mg was weighted in duplicate and sealed hermetically into Tzero pans (TA Instruments). Samples were equilibrated at −80 °C, then were heated at least 20–30 °C higher than T_g_ at a heating rate of 20 °C/min, then quickly cooled to −80 °C at a cooling rate of 10 °C/min. A second heating step was applied by heating the samples to 140 °C at a rate of 20 °C/min. T_g_s of the samples in pans containing pin holes to allow moisture to escape were measured using a TA Q2000 DSC equipped with a refrigerated cooling accessory (TA Instruments, New Castle, DE, USA). Samples (7–12 mg weighed into the pans with pin holes) were equilibrated at −30 °C. The samples were then heated to 100 °C, at a heating rate of 20 °C/min, followed by cooling to −30 °C, at a cooling rate of 10 °C/min. A second heating step was applied at a rate of 20 °C/min. The onset T_g_ of the second heating was recorded as the T_g_ using TRIOS software v5.1.1 (Universal Analysis), unless otherwise stated. A tangent was drawn on the straight line of the second heating step prior to baseline shift occurred to the endothermic direction. Then, a second tangent was drawn on the slope, followed by calculation of the cross point using TRIOS software v5.1.1.

### 2.8. Statistical Analysis

Statistical analysis was conducted using SAS Software Version 9.4 (SAS Institute, Cary, NC, USA). The significant differences in T_g_ between solid dispersions were determined using Tukey’s multiple comparison test at alpha 0.05.

## 3. Results and Discussion

### 3.1. Long Term Physical Stability of Citric Acid Amorphous Solid Dispersions Towards Crystallization Measured by PXRD

**Figure 1:**
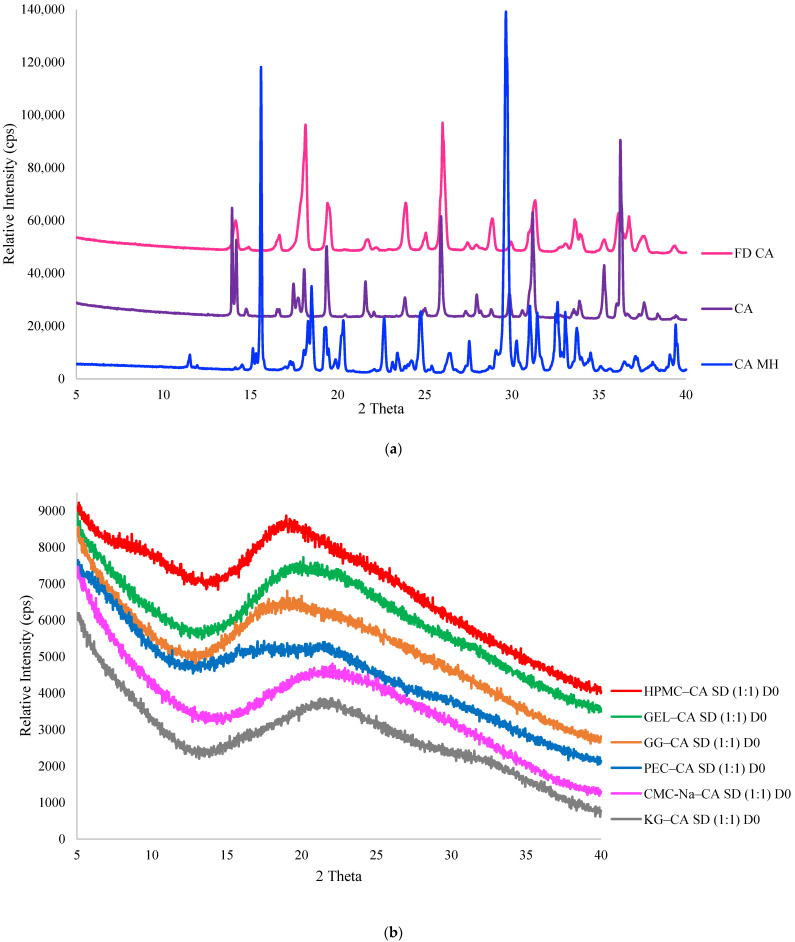
PXRD patterns of: (**a**) Anhydrous citric acid (CA), citric acid monohydrate (CA MH), and lyophilized citric acid (FD CA) showing evidence of crystallinity; and (**b**) 1:1 CA–polymer solid dispersions (SD) at day 0 showing evidence of amorphous structure.

The physical forms of CA (citric acid) and FD CA (freeze dried citric acid) were determined from their PXRD patterns. As expected, the initial CA was found to be in a crystalline state (Figure 1a), and the PXRD pattern indicated it was in an anhydrous crystalline form [21]. Lyophilization of CA alone in solution did not produce an amorphous form, as anticipated from its low T_g_ value and its high crystallization tendency at temperatures above its T_g_ [25]. Moreover, the T_g_′ (T_g_ of the maximally freeze concentrated solution) of CA (−55.1 °C, [38]) was below the temperature used during lyophilization, and compounds dried at temperatures above T_g_′ are prone to collapse [39]. The PXRD patterns of CA and FD CA exhibited slight differences in terms of peak intensities, peak shapes, peak widths, and peak multiplicity (Figure 1a). This raised the question of whether the residual water remaining in the structure after lyophilization could lead to the formation of CA monohydrate, and how comparable the crystalline structure of CA is to that of CA monohydrate. To address this, the PXRD pattern of FD CA was also compared to that of CA monohydrate. From the comparison of diffractograms, it was observed that the PXRD peak positions, intensities, and shapes differed significantly between the FD CA and CA monohydrate, indicating that their crystalline structures were not equivalent, and the FD CA diffractogram was more similar to that of anhydrous CA than CA monohydrate. For instance, PXRD patterns for CA monohydrate exhibited distinct, high-intensity peaks at 15.6° and 29.66° 2θ, which were absent in both CA anhydrous and FD CA (Figure 1a). Additionally, a very characteristic and intense peak at 36.22° 2θ present in CA PXRD patterns was also observed in FD CA but with a lower intensity. This peak was not present in CA monohydrate diffractograms. These findings clearly indicated that FD CA did not lead to the formation of CA monohydrate upon lyophilization. The difference in PXRD patterns of CA and FD CA was ascribed to some disorder, smaller particle size, and/or residual water present in the crystalline lattice of FD CA following lyophilization.

The presence of amorphous ingredients, such as hydrocolloids, in a formulation may create conditions favorable for amorphization of a co-formulated crystalline ingredient during processing (e.g., lyophilization or spray-drying), often by elevating the T_g_ of the system compared to the T_g_ of the target compound alone and/or by stabilizing the amorphous form via favorable intermolecular interactions that inhibit crystallization [34,35]. Amorphization will not occur spontaneously by simply blending amorphous polymers with crystalline ingredients when the process does not disrupt the initial crystal matrix. The ratio of ingredients in a formulation influences the resulting form and stability of the product. The amount of polymer needed to produce amorphous solid dispersions of CA varied among the polymers studied. Less PEC or CMC-Na (20% *w*/*w*) was needed to amorphize CA than GEL, GG, or HPMC (30% *w*/*w*) (Figure S1a–f). KG was the polymer that required the highest amount (40%) to yield amorphous CA in the solid dispersion (Figure S1f). When CA crystallized in the polymer dispersions during lyophilization, its PXRD pattern was that of the anhydrous crystalline form.

Amorphous solid dispersions of CA in a 1:1 ratio with all polymers were successfully prepared, as shown in Figure 1b, and these dispersions were subjected to a controlled temperature and RH storage stability study. Significant differences were found in the physical stabilities of the different 1:1 polymer–CA amorphous dispersions during storage (Table 1). All dispersions remained amorphous at 0% RH and both temperatures for the duration of the study (Figure S2a,b). As RH increased to 32%, CA crystallized from some polymer dispersions but not others. For instance, CA crystallized in the KG–CA dispersions within three weeks at 32% RH at both temperatures but remained amorphous in the CMC-Na–CA and PEC–CA dispersions at this RH (Table 1). Storage temperature had a more pronounced effect for the HPMC–CA, GG–CA, and GEL–CA dispersions stored at 32% RH than those stored at 0% RH. For example, at 32% RH, CA crystallization was evident at 28 days for the GG–CA and HPMC–CA dispersions, and at 63 days for GEL–CA at 40 °C (Table 1), whereas CA remained amorphous in these dispersions throughout the storage study at the lower temperature. Following the onset of CA crystallization evident in PXRD patterns, the intensity of the peaks in the PXRD did not change over longer storage periods (Figure S2c). At a higher RH (54%), CA crystallization occurred sooner (Table 1). CA crystallized in the KG and GG dispersions within 14 days, and in 21 days in PEC and HPMC dispersions, at 54% RH and both temperatures. CA remained amorphous longer in the CMC–CA dispersions at 54% RH, with the onset of CA crystallization occurring at 63 days at 25 °C, and at 49 days at 40 °C. Based on both the minimum amount of polymer needed to produce amorphous CA, as well as the stability of the amorphous CA in the 1:1 polymer dispersions during storage, the ranked ordering of polymer performance from most effective to least effective was as follows: CMC-Na > PEC > GEL > HPMC > GG > KG.

### 3.2. FTIR Spectroscopic Investigation of Interactions Between Citric Acid and Polymers

**Figure 2:**
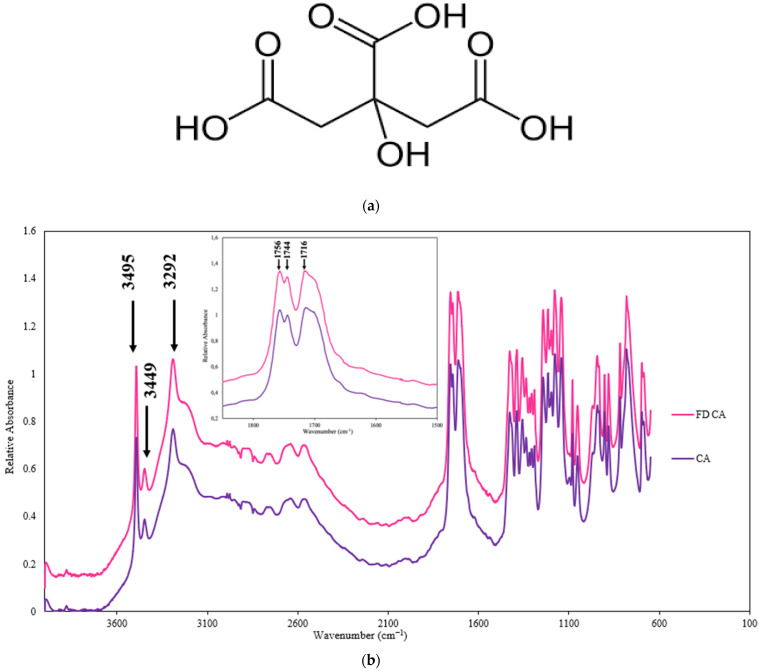
(**a**) Chemical Structure of citric acid (CA); (**b**) Mid-infrared spectra of crystalline citric acid samples, before (CA) and after lyophilization (FD CA). The NH/OH and carbonyl stretching regions are shown.

CA is a tricarboxylic acid containing three carboxylic functional groups, two of which are equivalent, and one hydroxyl group which is located at the center of the molecule (Figure 2a). These functional groups of CA can be involved in intermolecular hydrogen bonding: -OH_(alc)_ and -OH_(ac)_ are both hydrogen bond donors (HBDs) and acceptors (HBAs), and carbonyl carboxylic acids are HBAs. Thus, CA is composed of four potential HBD and seven potential HBA groups. As a result of strong intermolecular interaction via hydrogen bonding, carboxylic acids generally exist as dimers [40]. Dimerization of CA through hydrogen bonding between the carboxylic groups has been reported previously [41,42,43]. The strong intermolecular interaction between two monomers of CA needs to be overcome to be able to form intermolecular hydrogen bonding between CA and any other compound, and polymers which form more favorable intermolecular interactions with CA would therefore be anticipated to form more stable amorphous solid dispersions with CA in processes that disrupt the initial CA crystalline structure.

There are six intense bands in the IR spectrum of CA that correspond to the CA dimer structure: at 3498 cm^−1^, 3291 cm^−1^, 1756 cm^−1^, 1708 cm^−1^, 1174 cm^−1^, and 1140 cm^−1^ [43]. If these peaks disappear in the IR spectra of the CA–polymer solid dispersions, it could be attributed to the breaking of hydrogen bonding between two CA monomers and thus a new interaction formation between CA and a polymer. Intermolecular hydrogen bonding formation between compounds are highly dependent on available functional groups for hydrogen bonding as well as their potential HBA and HBD strengths. Therefore, the relative HBA and HBD strengths of the functional groups in CA and polymers were determined based on the pK_BHX_ scale [44], as shown in Table 2. These traits are taken into consideration in later discussions of polymer effectiveness at delaying the recrystallization of citric acid.

The interactions between CA and polymers via hydrogen bonding were investigated using FTIR, with emphasis on the peak shape and location in the hydroxyl and carbonyl regions of the spectra. As a control, the FTIR spectra of CA and FD CA were compared (Figure 2b) and found to have almost identical peak shapes and locations. Strong bands were found at 3495 cm^−1^, 3449 cm^−1^ and 3292 cm^−1^ in the hydroxyl region of the IR spectrum of CA (Figure 2b). These bands correspond to the alcohol and carboxylic OH groups of CA. Moreover, the IR spectra of CA had three bands in the 1800–1600 cm^−1^ region, at 1756 cm^−1^, 1744 cm^−1^, and 1716 cm^−1^ (Figure 2b), attributed to the stretching of the C=O bond of the carboxyl groups in CA.

**Figure 3:**
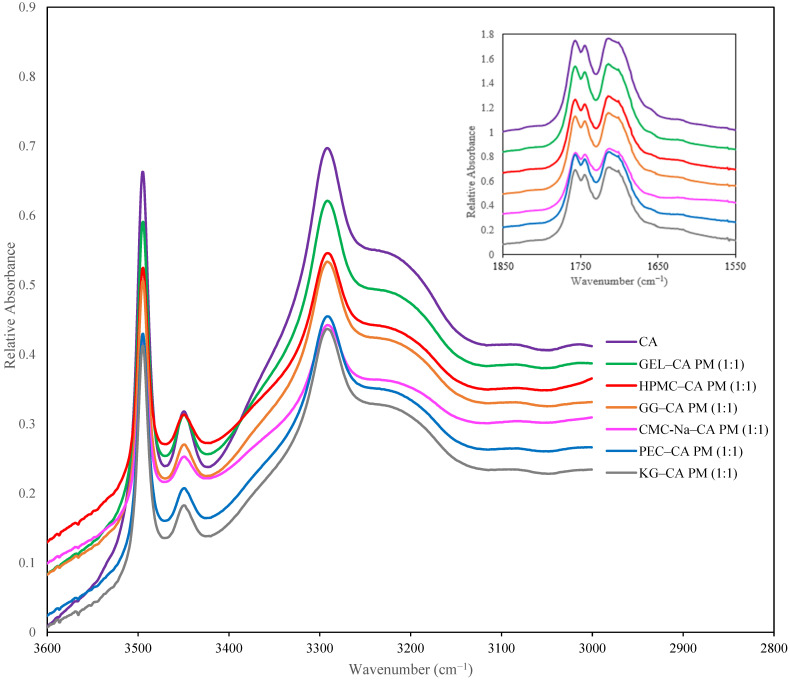
Mid infrared spectra of 1:1 Citric Acid (CA)–Polymer physical mixtures (PMs).

**Figure 4:**
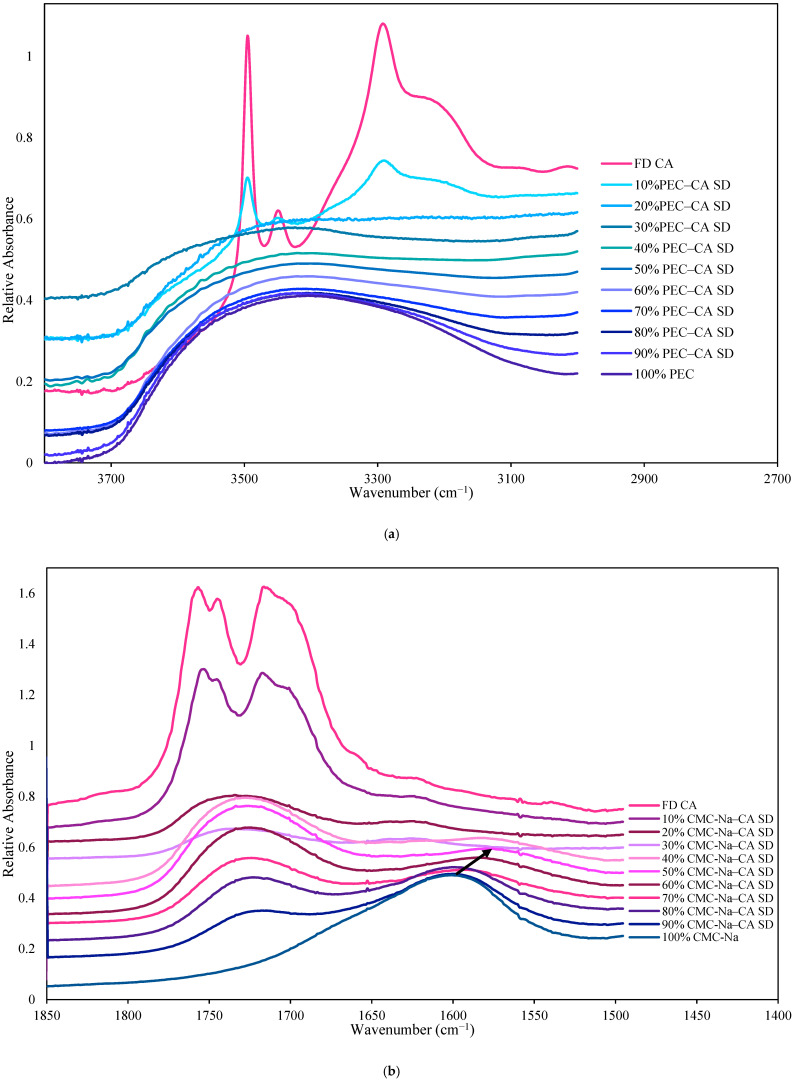

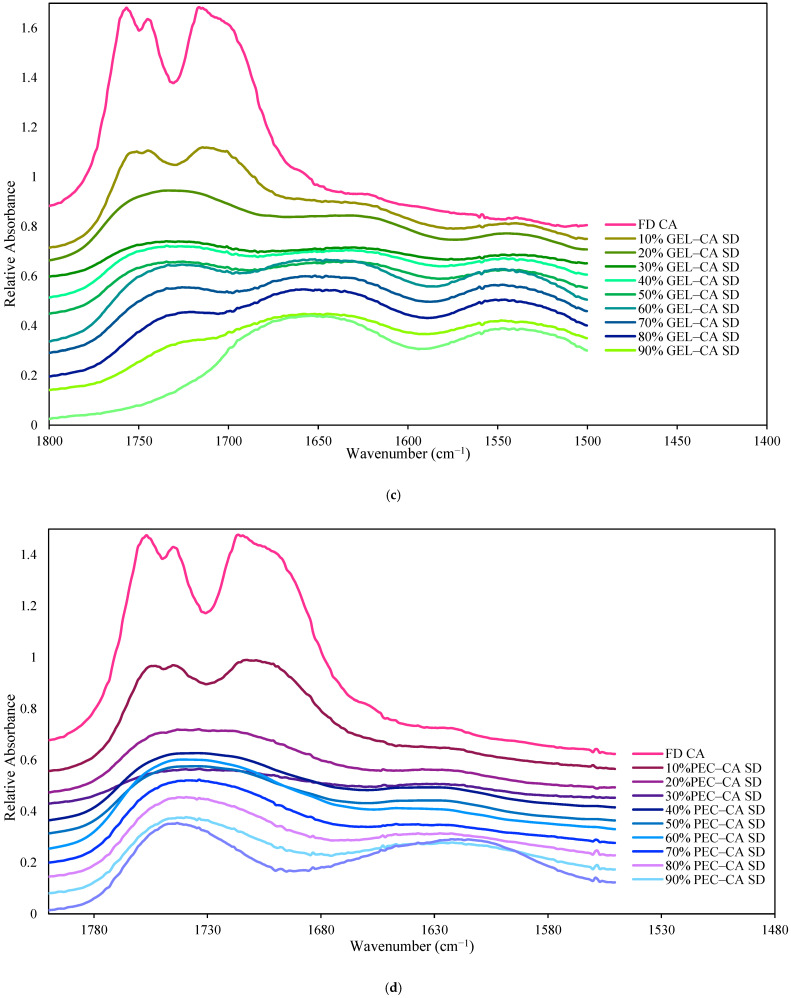
Mid-infrared spectra of CA–Polymer solid dispersions (SDs) made with different ratios shown as follows: (**a**) the NH/OH region of the CA–PEC SDs; the carbonyl stretching region of (**b**) CA–CMC-Na; (**c**) CA–GEL; and (**d**) CA–PEC SDs.

When polymers were simply blended with CA at a 1:1 ratio, the IR spectra of the physical mixtures resembled that of CA in terms of peak shapes and positions, as shown in Figure 3, documenting the lack of interaction between CA and the polymers. This was not the case for the lyophilized CA–polymer solid dispersions (Figure 4, Figures S3 and S4). Changes in peak locations and/or shapes in the IR spectra of the solid dispersions were found, when compared to the spectra of both the individual polymers and the FD CA and were highly associated with the polymer to CA ratio. We start by first considering the spectra of the polymers (Figure 4, Figures S3 and S4). A broad single band, which non-crystalline polymers tend to contain [40], was found in the hydroxyl regions of the IR spectra of the polymers, with maximum absorbance detected at 3400 cm^−1^, 3419 cm^−1^, 3322 cm^−1^, 3461 cm^−1^, 3388 cm^−1^, and 3422 cm^−1^ for CMC-Na, PEC, GEL, HPMC, GG, and KG, respectively (Figure 4a and Figure S3a–c). In addition, peaks in the carbonyl region occurred at 1600 cm^−1^ for CMC-Na, 1653 cm^−1^ and 1558 cm^−1^ for GEL, and 1743 cm^−1^ and 1622 cm^−1^ for PEC (Figure 4b–d). It should be noted that HPMC, KG, and GG do not contain any carbonyl groups, which was reflected in their spectra (Figure S4a–c).

When amorphous CA–polymer dispersions were formed, peak shifts in the hydroxyl region of the FTIR spectra were found relative to the individual polymers. In pectin dispersions (CA–PEC 1:1), a peak shift to a lower wavenumber (3400 cm^−1^) relative to the maximum absorbance of PEC in the hydroxyl region (3419 cm^−1^) was present, attributed to the hydrogen bonding between CA and PEC (Figure 4a and Table 3). Larger peak shifts to lower wavenumbers in the hydroxyl region relative to the polymers were found when the CA–HPMC and CA–KG amorphous solid dispersions were formed (Table 3 and Figure S3a,b): shifts of 40 cm^−1^ and 38 cm^−1^, respectively. By contrast, peak shifts to lower wavenumbers were not present in the spectra of CA dispersions with CMC-Na, GEL, and GG compared with the spectra of the individual polymers, although evidence of intermolecular hydrogen bonding was found when comparing the dispersion spectra to that of crystalline CA (Table 3 and Figure S3c).

The crystalline form of CA exhibited three main peaks (3495 cm^−1^, 3449 cm^−1^, and 3292 cm^−1^) in the hydroxyl region of the FTIR spectra. When amorphization of the CA was successful in the polymer dispersions (as evidenced by PXRD diffractograms), these individual peaks in the CA spectra were no longer present and instead a broad peak in the hydroxyl region was found. The disappearance of the individual peak characteristics of intramolecular CA hydrogen bonding, such as in dimers, provided evidence of disruption of the CA crystalline structure. For example, the FTIR spectra of the hydroxyl stretching region of the CA–CMC-Na dispersions are shown as a function of the polymer concentration in Figure S3c. Similar peaks were present in the spectra of the FD CA and the 10% CMC-Na–CA dispersion, indicative of the presence of crystalline CA in the polymer dispersion, which was confirmed by PXRD. Increasing the polymer concentration in the dispersion to 20% or more resulted in amorphous CA solid dispersions, evidenced by the PXRD spectra, the loss of the IR spectra peaks characteristic of crystalline CA, and broad peak shape alteration as polymer concentration increased, the latter indicative of reduced intermolecular CA interactions and increased intramolecular interactions between the CA and the CMC-Na polymer. Similar trends were found in the hydroxyl region of the spectra of the solid dispersions made with the other polymers (Figure 4a and Figure S3), although the amount of polymer needed to disrupt the presence of crystalline CA peaks varied.

Peak shifts in the carbonyl region of the FTIR spectra of the CA–polymer dispersions using CMC-Na, PEC and GEL polymers were also indicative of intermolecular interactions of hydrogen-bonded carbonyl groups in the dispersions (Table 3 and Figure 4b–d). The carbonyl peak of CMC-Na at 1600 cm^−1^ shifted to 1575 cm^−1^ in the CMC-Na–CA (1:1) solid dispersion. Similarly, the carbonyl peaks of GEL at 1653 cm^−1^ and 1558 cm^−1^, shifted to 1634 cm^−1^ and 1546 cm^−1^, respectively, in the CA–GEL dispersion (Table 3 and Figure 4c). Lastly, one of two carbonyl peaks of PEC (1743 cm^−1^) shifted to 1731 cm^−1^ (Table 3 and Figure 4d), which could be attributed to more favorable hydrogen bonding between PEC and CA. These three polymers produced the most stable amorphous CA in the polymer dispersions (Table 1), indicating the potential of the important role of intermolecular hydrogen bonding between CA and polymer carbonyl groups on stabilizing the amorphous dispersion.

Based on the structural interpretation of citric acid and the polymers, as well as their interactions in the dispersions, it can be concluded that polymers containing carbonyl groups (CMC-Na, PEC, and GEL) were shown to be more effective in stabilizing the amorphous solid dispersions under low RH% storage conditions (32% RH) than polymers lacking carbonyl groups (KG, GG, and HPMC). This latter group of polymers could have been more prone to destabilize the amorphous solid dispersions by interacting with water, rather than with citric acid. This phenomenon could have led to a weakening of the hydrogen bonding interaction between the polymers and citric acid, ultimately resulting in the crystallization of citric acid from the amorphous dispersions (Table 1).

### 3.3. Potential Ionic Interaction Between Citric Acid and Select Polymers

In addition to the intermolecular hydrogen bonding formation, the potential for ionic interactions between CA and some of the polymers in solid dispersions was considered. Citric acid is composed of three carboxylic acids with p*K*as of 3.1, 4.8, and 6.4 [45]. The pHs of individual polymers and CA in solution were measured as 7.21 for CMC-Na, 4.91 for GEL, 3.54 PEC, 8.18 for KG, 5.79 for HPMC, 6.24 for GG, and 2.33 for CA. Upon mixing CA with the polymers, the pHs of the solutions changed to 3.01 for CMC-Na–CA, 2.42 for GEL–CA, 2.39 for PEC–CA, 2.37 for KG–CA, 2.32 for HPMC–CA, and 2.33 for GG solid dispersions. Thus, the pHs of the CA–polymer solutions were below the p*K*a of CA before lyophilization. The speciation curve of citric acid (prepared based on the first p*K*a of 3.1) is shown in Figure S4d. The fraction of protonated species in CA at pHs 2.3, 2.4, and 3.0 was calculated to be 0.87, 0.85, and 0.58, respectively. Therefore, the highest fraction of deprotonated CA was present in the CMC-Na–CA dispersions.

The carboxylic acids found in the structure of CA can react with metals, which may result in the formation of carboxylate salts on the metal. In this study, the sodium portion (metal) of CMC-Na could be attracting the carboxylate anion of CA which could lead to the formation of an ionic network. GEL, composed of amino acids which carry both negative and positive charges, also has the potential to interact with CA via ionic interactions between the amine groups in GEL and the ionized carboxylic acid group of CA. PEC, a partially methoxylated polygalacturonic acid polymer, is generally believed to have a pKa in the range between pH 3.5–4.5 [46], and therefore would also be able to participate in some ionic interactions with CA. Ionic interactions may be stronger than hydrogen bonding [47], and CMC-Na, GEL, and PEC polymers have the potential to stabilize amorphous CA solid dispersions by both intermolecular hydrogen bonding and ionic interactions. It was these three polymers that resulted in the most stable amorphous CA in the dispersions (Table 1).

### 3.4. Moisture Sorption Isotherm Profiles

Water is ubiquitous; therefore, exposure of ingredients and products to moisture is unavoidable during handling, processing, and storage. Understanding how water influences the stability of products is important for designing formulation and packaging strategies for optimizing quality and shelf-life.

**Figure 5:**
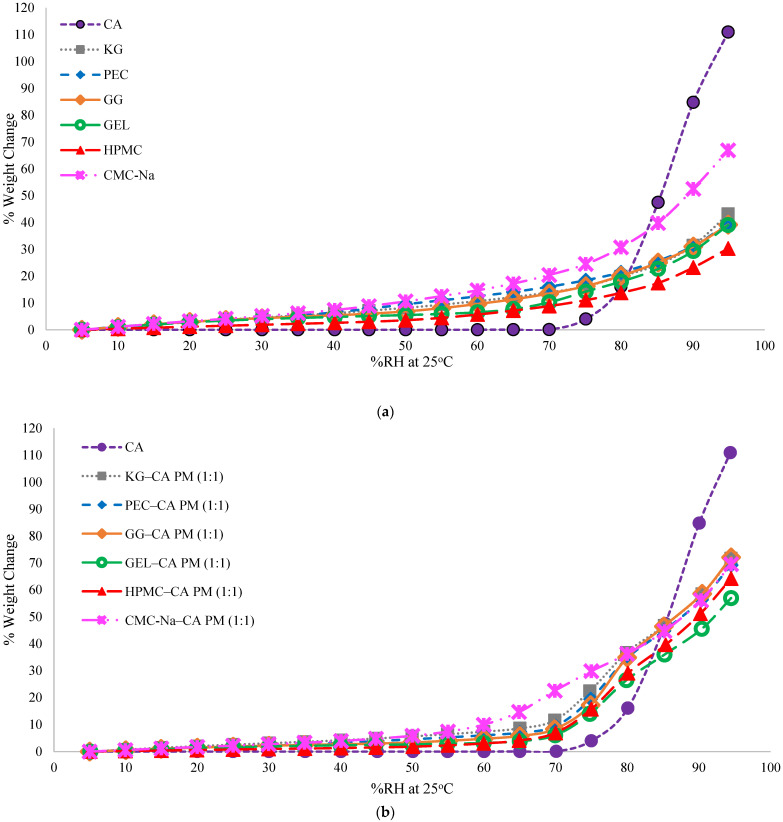

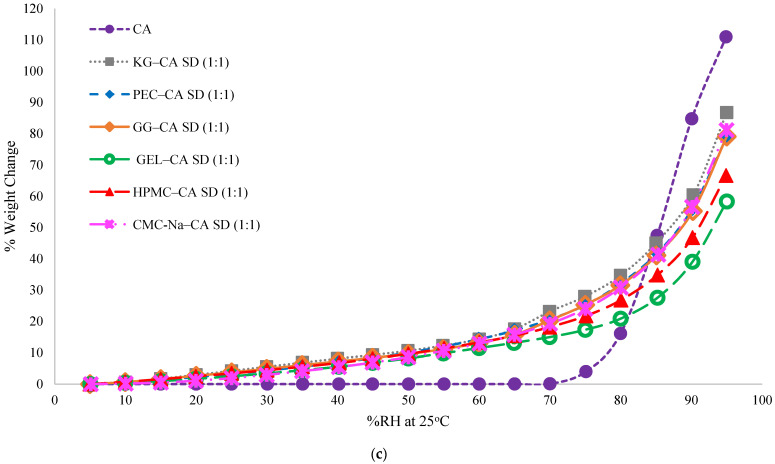
Moisture sorption profiles of samples at 25 °C: (**a**) CA and polymers; (**b**) 1:1 CA–polymer physical mixtures (PMs); (**c**) 1:1 CA–polymer solid dispersions (SDs).

The moisture sorption profiles of individual CA and polymer ingredients, physical mixtures, and solid dispersions were collected at 25 °C (Figure 5a–c) and 40 °C (Figure S5a–c). The moisture sorption profiles of crystalline CA and FD CA were also generated (Figure S6a). Anhydrous crystalline CA has a deliquescence RH of 74–75% RH at 25 °C, and CA monohydrate has a deliquescence RH of 78% RH [21,48]. Deliquescent crystals exhibit little moisture sorption below their deliquescence RHs, limited to surface adsorption and capillary condensation [49]. Both CA and FD CA exhibited little moisture sorption below 75% RH, some moisture uptake at 75% RH, and increasing amounts of moisture sorption at higher RHs, characteristic of anhydrous crystalline CA moisture sorption patterns. The higher hygrocapacity of FD CA was attributed to the smaller particle sizes and disorder resulting from lyophilization, which could increase the rate of moisture sorption. The polymers exhibited typical type II (sigmoidal) moisture sorption patterns (Figure 5a), characteristic of amorphous materials. All polymers sorbed more moisture than CA at RHs < 80% RH. Of the polymers, CMC-Na sorbed the most moisture (had the highest hygrocapacity), and HPMC sorbed the least (Figure 5a).

Moisture sorption profiles of the CA–polymer physical mixtures were compared to those of the solid dispersions (Figure 5b,c). The trends in relative moisture sorption exhibited by the individual polymers did not generally carry forward to the trends in the physical mixtures or dispersions. While CMC-Na was the most hygroscopic polymer and resulted in the highest moisture sorption in physical mixtures, between 60% and 80% RH, other polymer physical mixtures sorbed more water at higher RHs, and other solid dispersions sorbed more water across a wider RH range. Gelatin, not HPMC, produced the least hygroscopic physical mixtures and solid dispersions.

Solid dispersions sorbed more moisture than physical blends at low RHs (<75% RH), attributed to the presence of amorphous CA. The magnitude of the difference in moisture uptake between solid dispersions and physical blends was the lowest for CMC-Na–CA (the most hygroscopic polymer) and the highest for HPMC–CA (the least hygroscopic polymer). For instance, HMPC–CA solid dispersions sorbed 10% (*w*/*w*) more moisture than the counterpart physical blends at 60% RH; whereas CMC-Na–CA solid dispersion sorbed only 3% (*w*/*w*) more moisture than the physical blends at that RH (Figure 5c).

**Figure 6:**
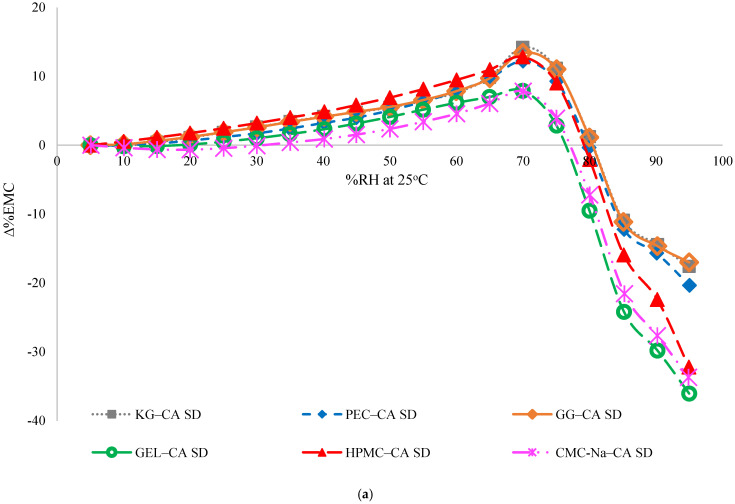

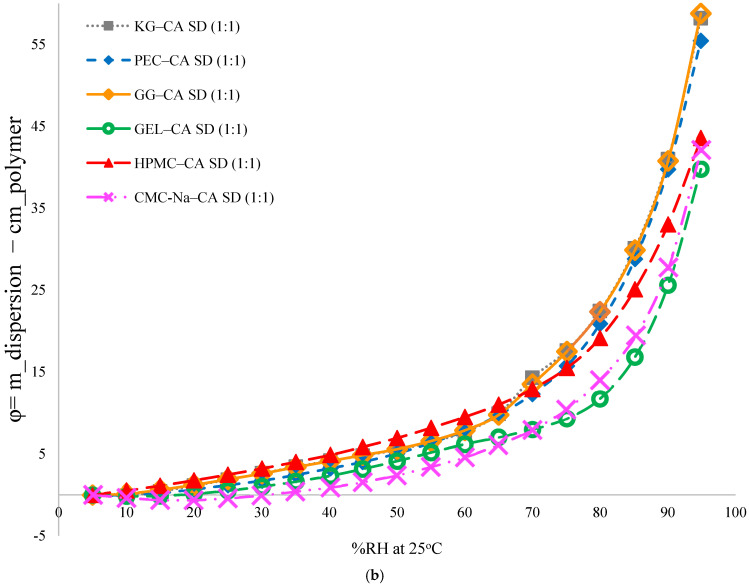
(**a**) Percent equilibrium moisture content (∆% EMC) versus %RH of all the solid dispersions (SD); (**b**) Difference between polymer and dispersion moisture sorption.

Predicted moisture sorption profiles for the solid dispersions were calculated based on an additive model in which the contribution of individual lyophilized ingredients (FD CA and polymers) for the moisture sorption is taken into account. Moisture sorption was considered to be synergistic when experimental samples sorbed more moisture than the calculated values based on the additive model. Experimental and predicted moisture sorption profiles of the solid dispersions and the difference between additive model and experimental values are shown in Figure 6a and Figure S6b–f. These differences increased as RH% increased from 0% to 70% RH, attributed to the amorphous CA sorbing more moisture than crystalline CA below the deliquescence RH. The largest differences (∆% EMC) were found in the KG–CA and HPMC–CA solid dispersions at 0–70% RH, while CMC-Na–CA solid dispersions had the smallest differences in the same conditions (Figure 6a). As RH increased above 75% RH, the trends in ∆% EMC changed, with the experimental samples sorbing less moisture than the predicted model, driven by the higher amount of water sorbed by crystalline CA above its deliquescence RH. The impact of amorphous CA on the moisture sorption of solid dispersions was investigated by calculating the φ value (using Equation (1)), which eliminates the contribution of the polymers to the amount of moisture absorbed, in order to reveal the effect of CA only (Figure 6b). Positive φ values were found for the solid dispersions, meaning that CA contributed to the moisture sorption of the solid dispersions. The differences in φ values between the dispersion types, which all contained the same amount of CA and polymer, indicate that interactions between the CA and the polymers had an influence on moisture uptake. Otherwise, all φ values would have been similar. Trends in φ values based on polymer type varied above and below 70% RH.

There was no direct correlation found between the physical stability of the amorphous solid dispersions and the hygrocapacity of the individual polymers or solid dispersions. Based on the PXRD results, CMC-Na was the most effective polymer for inhibiting the recrystallization of amorphous CA, even though it was the most hygroscopic polymer used in this study. The crystallization inhibitor ability of the polymers varied across RH% conditions and was not related to the amount of moisture sorbed at these conditions. For example, the amount of moisture sorbed by the KG–CA dispersion (6.8% *w*/*w*) and the GG–CA solid dispersion (6.1% *w*/*w*) were similar at 35% RH (Figure 5c); however, the KG–CA dispersion crystallized in 21 days at 32% RH and 25 °C, while the GG–CA dispersion remained amorphous for more than 189 days in this environment (Table 1).

### 3.5. Glass Transition Temperatures

The onset T_g_s of amorphous CA and the polymers studied were as follows: CA, 11 °C [25]; CMC-Na, 191 °C [50]; KG, 161 °C [51]; GEL, 151 ± 3 °C; HPMC, 145 °C [33]; GG, 108 °C [52]; and PEC, 90 ± 2 °C. All polymers had higher T_g_s than CA and thus were candidates for increasing the physical stability of amorphous CA based on the theory that increases in T_g_ slows molecular mobility and delays crystallization [53].

For determining the T_g_s of the CA–polymer dispersions, DSC measurements were conducted both in the absence and in the presence of a pinhole in the pans for the CA–polymer amorphous solid dispersions on the day when the samples were withdrawn from the freeze dryer (Table 4; Figures S7a–f and S8a–f). Lower T_g_s were found when the measurement was conducted without a pin hole, attributed to water remaining in the samples that acted as a plasticizer (water has a low T_g_: 135 K [54]). The highest T_g_ was found for CMC-Na–CA (14.3 °C), and the lowest for KG–CA (−10.4 °C). All of these T_g_s were below the temperatures used during storage and analysis in this study. When a pinhole was introduced into the pans to enable the escape of water, the measured T_g_s ranked in the order of highest to lowest as follows: CMC-Na–CA (49.5 °C) > GEL–CA > PEC–CA > KG–CA > GG–CA > HPMC–CA (16.6 °C). Regardless of whether or not a pinhole was used during DSC analyses, the T_g_ of solid dispersions did not follow the same trend order as the T_g_ of the individual polymers, indicative of intermolecular interactions between the polymer and CA. While CMC-Na and GEL had the highest T_g_s of the polymers studied, and produced dispersions with the highest T_g_s (which would be expected based on Fox and Gordon-Taylor models [55,56], both with and without a pinhole, HPMC had among the highest polymer T_g_s but produced dispersions among the lowest T_g_s, and PEC had the lowest T_g_ of the polymers but produced dispersions with intermediate T_g_s (Table 4). The specific heat capacities (Cps) of the samples were also determined from the DSC scans (Figures S7a–f and S8a–f), with the thought that the intermolecular interactions between the polymers and CA that altered the vibration, rotation, and translation of the molecules and therefore Cp values would also have a role in stabilizing amorphous solid dispersions. However, the DSC instruments had not been specifically calibrated for this analysis and variations in Cp trends between samples run in hermetically sealed pans and pin-hole pans were found, which cautioned further use of these results. Future experiments could be designed to determine the Cp and enthalpy of the polymer–CA dispersions to better understand how the intermolecular interactions and thermal stability of the dispersions influence CA amorphization and stability.

Although CA solid dispersions were stored in temperature conditions above their T_g_s measured after lyophilization, the samples maintained their amorphous structures at 0% and 32% RHs at 25 °C (except for the KG–CA dispersions at 32% RH, attributed to the lower T_g_ of these dispersions) throughout the time scale of the experiment (189 days). A similar observation was reported by Hoppu et al. (2006), where amorphous dispersions of citric acid with paracetamol present in the rubbery state remained amorphous for at least 27 weeks in dry conditions [24]. Not only did the amorphous CA remain stable in these dispersions at temperatures above the T_g_ of both the individual CA and the dispersion, but the trends in stability did not follow the assumption that the polymer with the highest T_g_ could be the best crystallization inhibitor. Based on dry polymer T_g_, the expected ranked order for the stability of CA amorphous solid dispersions would be as follows: CMC-Na > KG > GEL > HPMC > GG > PEC. Based on the measured dispersion T_g_ (no pinhole, Table 4), the stability of amorphous CA dispersions would be as follows: CMC-Na > GEL > PEC > HPMC > GG > KG. The delay in CA crystallization from the dispersions in the storage study (Table 1) was documented to be as follows: CMC-Na > PEC > GEL > HPMC > GG > KG. This is quite similar to the T_g_ of the dispersions measured without a pinhole, therefore accounting for the water present in the matrix. Interestingly, PEC had the lowest T_g_ of the polymers, produced a dispersion with an intermediate T_g_, and resulted in a dispersion with the second highest stability in terms of delaying CA crystallization. There are reports that the formation of hydrogen bonding and/or ionic interactions among the compounds of the dispersion being a more important contributor to physical stability than T_g_ [35,36,57]. This study indicates that more than T_g_ is influencing the stability of the amorphous CA dispersions, and that CA crystallization is delayed to different extents in polymer dispersions in environmental conditions exceeding the T_g_ of both CA and the dispersion.

## 4. Conclusions

Amorphization of CA was successful in the presence of polymers. Citric acid solid dispersions maintained their amorphous characteristics for more than 189 days of storage at 0% RH, 25 °C and 40 °C, regardless of the polymer type used. As storage RH increased to 32% and 54% RHs, the performance of the polymers as crystallization inhibitors varied, suggesting the following ranked order: CMC-Na > PEC ≥ GEL > HPMC > GG > KG. The T_g_s of CA amorphous solid dispersions were measured to be well below the temperatures used for the storage studies. Thus, all the polymers were able to inhibit the recrystallization of citric acid even from the rubbery state at dry conditions (0% RH and, in some cases, 32% RH). The polymers that enabled the most extensive ionic interaction (CMC-Na, PEC, and GEL) and intermolecular hydrogen bonding (as indicated by the FTIR results and based on the structural interpretation of HBD and HBA groups/strengths of CA and polymers) with citric acid were found to be the best stabilizers of amorphous citric acid. Polymers lacking carbonyl functional groups were at a disadvantage both in terms of the amount of polymer required to amorphize citric acid and in terms of the stabilizing of citric acid amorphous solid dispersions at different storage conditions. While polymer T_g_ and hygroscopicity were also contributing factors to the stability of the dispersions, they were not independent of intermolecular interaction. The findings of this study contribute to the understanding of physicochemical factors that stabilize amorphous CA in polymer matrices, which may be useful for designing other stable amorphous acid–polymer dispersions. In future investigations for comparative analysis, in silico molecular simulation techniques could be used to provide valuable insight into molecular dynamics in the stabilization of amorphous solid dispersions.

## Figures and Tables

**Table 1:** Interpretation of PXRD patterns of 1:1 Citric Acid (CA)–Polymer solid dispersions (SD) collected throughout a 189-day storage study in controlled temperature and RH conditions. PXRD patterns that contained sharp peaks were interpreted as having CA crystalline structures (C), those that had halo patterns were interpreted as being amorphous (A), and partially crystalline samples, in which onset crystallization of CA was started but peaks were present in few locations (2 Theta) with low intensity, were labeled as (PC). Different colors represent the differences in the solid structure of the dispersions.

RH (%) and T (°C)	CA–CMC-Na SD	CA–PEC SD	CA–GEL SD	CA–HPMC SD	CA–GG SD	CA–KG SD
0% RH- 25 °C	>189-A	>189-A	>189-A	>189-A	>189-A	>189-A
0% RH- 40 °C	>189-A	>189-A	>189-A	>189-A	>189-A	>189-A
32% RH- 25 °C	>189-A	>189-A	>189-A	>189-A	>189-A	<21-C
32% RH- 40 °C	>189-A	>189-A	<63-PC	<28-PC	<28-PC	<21-C
54% RH- 25 °C	<63-PC	<21-C	<49-C	<21-C	<14-C	<14-C
54% RH- 40 °C	<49-C	<21-C	<49-C	<21-C	<14-C	<14-C

**Table 2:** Characterization of hydrogen bond donor and acceptor groups of citric acid (CA) and polymers based on the p*K*_BHX_ scale published by Laurence et al. [44].

Compound	Hydrogen Bond Donor		Hydrogen Bond Acceptor	
	Group	Strength	Group	Strength ^a^
CA	Hydroxyl	Strong	Hydroxyl	Medium
	Carboxylic	Very Strong	Carboxylic	Medium ^b^
PEC	Hydroxyl	Strong	Hydroxyl	Medium
	Carboxylic	Very Strong	Carboxylic	Medium ^b^
			Ether	Medium
			Ester	Medium
GG	Hydroxyl	Strong	Hydroxyl	Medium
			Ether	Medium
KG	Hydroxyl	Strong	Hydroxyl	Medium
			Ether	Medium
			S=O of sulfate ester	Medium
HPMC	Hydroxyl	Strong	Hydroxyl	Medium
			Ether	Medium
CMC-Na	Hydroxyl	Strong	Hydroxyl	Medium
	Carboxylic	Very Strong	Carboxylic Ether	Medium Medium

^a^ Acceptor strength was determined by p*K*_BHX_ scale. The classification of the acceptor strength was weak < 0.5 < medium < 1.8 < strong < 3.0 < very strong (Laurence et al. [44]). ^b^ No values were found for carboxylic acid acceptor group (Laurence et al. [44]), but the acceptor strength of the carboxylic acid carbonyl group was regarded as being comparable to the ester carbonyl group.

**Table 3:** FTIR peak shifts observed in the amorphous CA–polymer solid dispersions compared to the polymer alone.

CA–Polymer Dispersion	%CA	Hydroxyl Region (3600–3000 cm^−1^)	Total Shift	Carbonyl Region (1800–1500 cm^−1^)		Total Shift	
CA–PEC	0 → 50	3419 → 3400	19	1743 → 1734	1622 → 1647	9	−25
CA–KG	0 → 50	3422 → 3384	38	N/A			
CA–GG	0 → 50	3388 → 3402	−14	N/A			
CA–GEL	0 → 50	3322 → 3335	−12	1653 → 1634	1558 → 1546	19	12
CA–HPMC	0 → 50	3461 → 3421	40	N/A			
CA–CMC-Na	0 → 50	3400 → 3400	0	1600 → 1575		25	

N/A: not applicable.

**Table 4:** Onset glass transition temperatures (T_g_) of citric acid–polymer solid dispersions determined using DSC and pans with or without a pinhole. Different superscript letters indicate significant differences within each column.

Dispersion Type	Onset T_g_ (°C) at (°C) Day 0 (Pin Hole)	Onset T_g_ (°C) at Day 0 (No Pin Hole)
CMC-Na–CA SD	49.5 ± 1.1 ^A^	14.3 ^a^
GEL–CA SD	44.4 ± 1.3 ^A^	13.1 ± 2.5 ^a^
GG–CA SD	21.8 ± 2.4 ^DC^	−9.5 ± 1.8 ^b^
HPMC–CA SD	16.6 ± 1.8 ^D^	−7.8 ^b^
KG–CA SD	24.7 ± 0.2 ^C^	−10.4 ± 0.1 ^b^
PEC–CA SD	33.5 ± 0.8 ^B^	−5.7 ± 0.2 ^b^

## Data Availability

The original contributions presented in this study are included in the article/Supplementary Material. Further inquiries can be directed to the corresponding author.

## Supplementary Materials

The following supporting information can be downloaded at: https://www.mdpi.com/article/10.3390/polym17030310/s1, Figure S1: PXRD patterns of various ratios of CA to polymer: (a) CA–PEC solid dispersion, (b) CA–CMC-Na solid dispersion, (c) CA–GEL solid dispersion, (d) CA–GG solid dispersion, (e) CA–HPMC solid dispersion, (f) CA–KG solid dispersion; Figure S2: PXRD patterns of: (a) 1:1 CA–polymer solid dispersions at 0% RH and 25 °C on day 189, (b) 1:1 CA–polymer solid dispersions at 0% RH and 40 °C on day 189, (c) 1:1 CA–GG solid dispersions; Figure S3: Mid infrared spectra of: (a) CA–HMPC solid dispersions (SD), (b) CA–KG solid dispersions (SD), (c) CA–CMC-Na solid dispersions (SD), made with various ratios (NH/OH region is shown); Figure S4: Mid infrared spectra of: (a) CA–GG solid dispersion (SD), (b) CA–HPMC solid dispersion (SD), (c) CA–KG solid dispersion (SD), made with various ratios, (carbonyl region is shown), (d) Speciation plot of CA; Figure S5: Moisture sorption profiles of samples at 40 °C: (**a**) CA and polymers, (**b**) 1:1 CA–polymer physical mixtures (PMs), (**c**) 1:1 CA–polymer solid dispersions (SDs); Figure S6: (a) Moisture sorption profiles of samples CA and FD CA at 25 °C. Experimental (EXP) moisture sorption profile of 1:1 CA–polymer solid dispersions (SD) vs. predicted (PRE) moisture sorption profile, (b) CA–PEC solid dispersions (SD) (c) CA–KG solid dispersions (SD), (d) CA–GEL solid dispersions (SD), (e) CA–GG solid dispersions (SD), (f) CA–HPMC solid dispersions (SD), (g) CA–CMC-Na solid dispersions (SD); Figure S7: DSC thermograms of solid dispersions without pin hole (The separate display of second scans is shown in the full scan graphs): (a) CA–CMC-Na solid dispersions (SD), (b) CA–GEL solid dispersions (SD), (c) CA–GG solid dispersions (SD), (d) CA–HPMC solid dispersions (SD), (e) CA–KG solid dispersions (SD), (f) CA–PEC solid dispersions (SD); Figure S8: DSC thermograms of solid dispersions with pin hole (The separate display of second scans is shown in the full scan graphs): (a) CA–CMC-Na solid dispersions (SD), (b) CA–GEL solid dispersions (SD), (c) CA–GG solid dispersions (SD), (d) CA–HPMC solid dispersions (SD), (e) CA–KG solid dispersions (SD), (f) CA–PEC solid dispersions (SD).
