# Supplementary material for: Impact of Polymer Physicochemical Features on the Amorphization and Crystallization of Citric Acid in Solid Dispersions
Source: Polymers (Basel). 2025 Jan 24;17(3):310. doi: 10.3390/polym17030310 (PMC11820854; doi:10.3390/polym17030310)
Supplement: Supplementary file 1 [file polymers-17-00310-s001.zip › polymers-3394712-supplementary.pdf]

a)

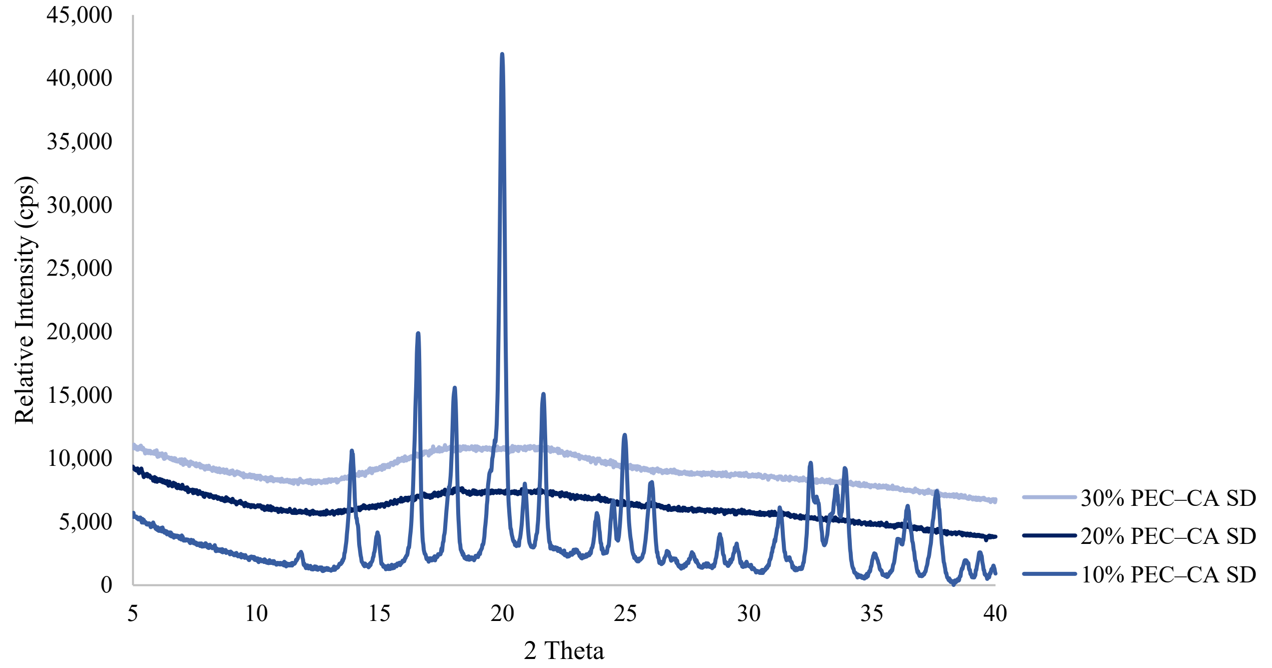
45,000
40,000
35,000
30,000
25,000
20,000
15,000
10,000
5,000
0
Relative Intensity (cps)
5
10
15
20
25
30
35
40
2 Theta
30% PEC–CA SD
20% PEC–CA SD
10% PEC–CA SD

b)

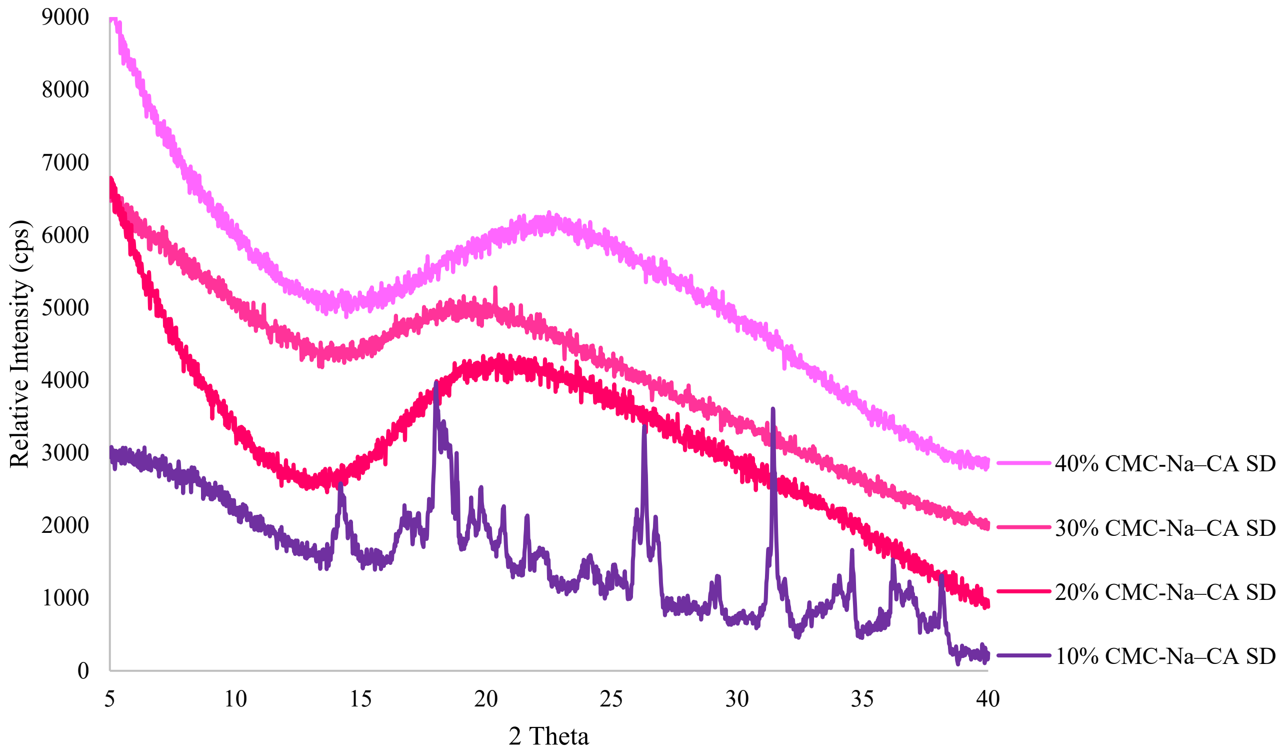
9000
8000
7000
6000
5000
4000
3000
2000
1000
0
Relative Intensity (cps)
5
10
15
20
25
30
35
40
2 Theta
40% CMC-Na–CA SD
30% CMC-Na–CA SD
20% CMC-Na–CA SD
10% CMC-Na–CA SD

c)

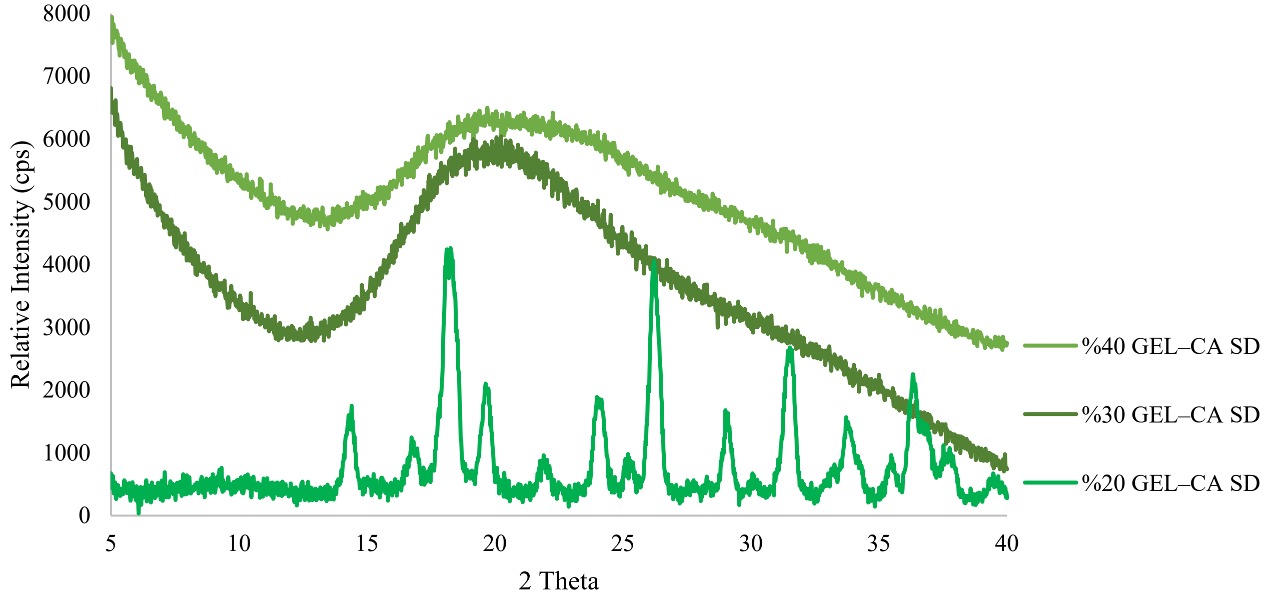
8000
7000
6000
5000
4000
3000
2000
1000
0
Relative Intensity (cps)
5
10
15
20
25
30
35
40
2 Theta
%40 GEL–CA SD
%30 GEL–CA SD
%20 GEL–CA SD

d)

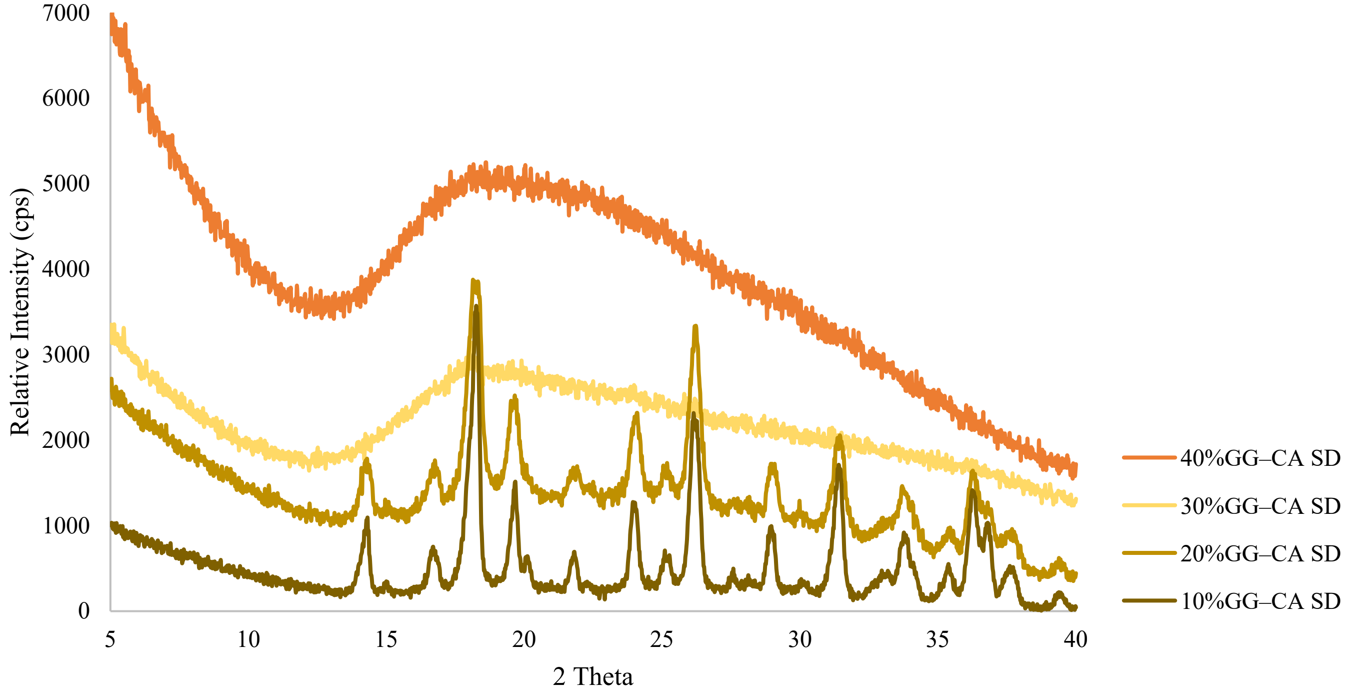
7000
6000
5000
4000
3000
2000
1000
0
Relative Intensity (cps)
5
10
15
20
25
30
35
40
2 Theta
40%GG–CA SD
30%GG–CA SD
20%GG–CA SD
10%GG–CA SD

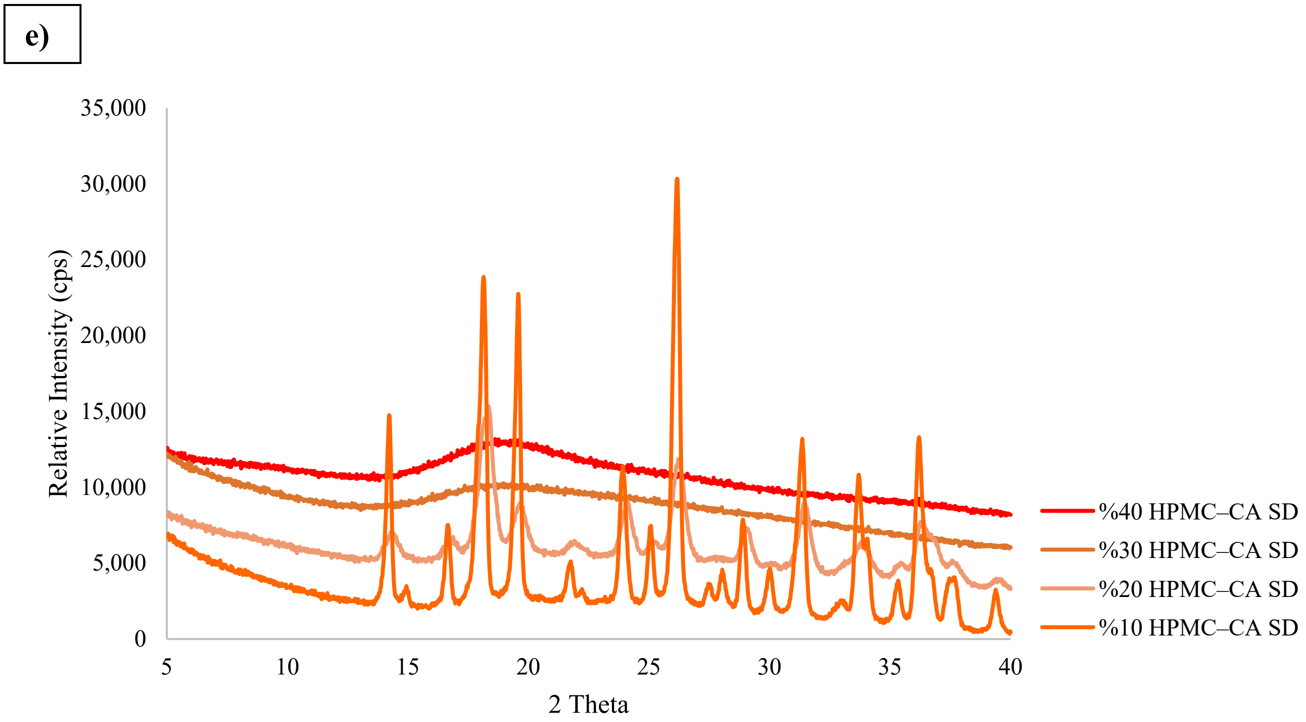

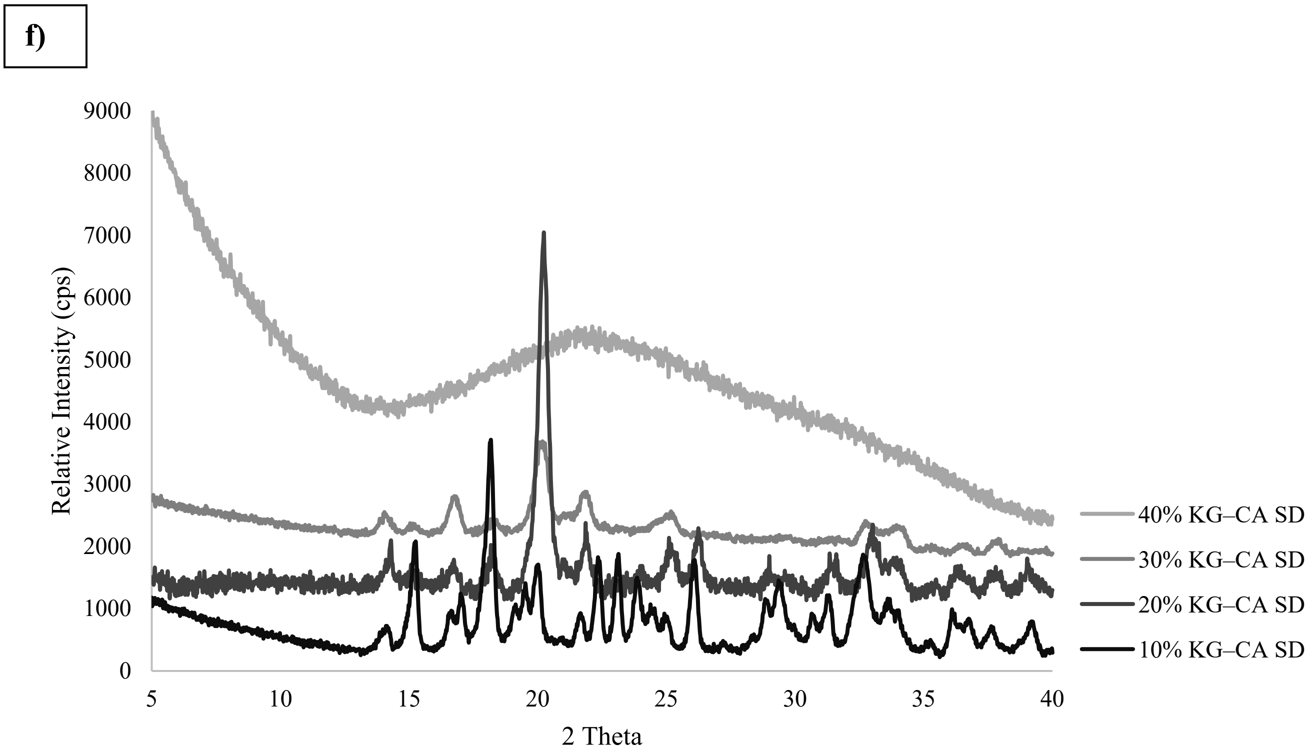

Figure S1: PXRD patterns of various ratios of CA to polymer: a) CA – PEC solid dispersion, b) CA – CMC-Na solid dispersion, c) CA – GEL solid dispersion, d) CA – GG solid dispersion, e) CA – HPMC solid dispersion, f) CA – KG solid dispersion.

a)

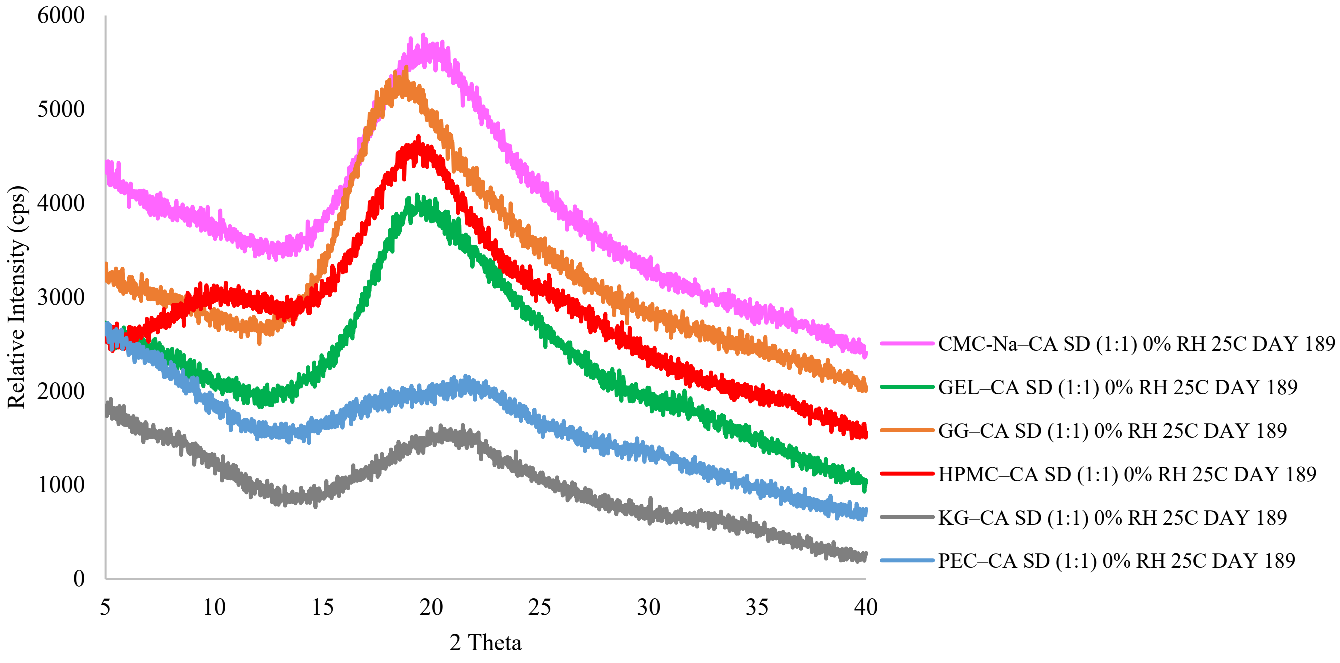
6000
5000
4000
3000
2000
1000
0
Relative Intensity (cps)
5
10
15
20
25
30
35
40
2 Theta
CMC-Na–CA SD (1:1) 0% RH 25C DAY 189
GEL–CA SD (1:1) 0% RH 25C DAY 189
GG–CA SD (1:1) 0% RH 25C DAY 189
HPMC–CA SD (1:1) 0% RH 25C DAY 189
KG–CA SD (1:1) 0% RH 25C DAY 189
PEC–CA SD (1:1) 0% RH 25C DAY 189

b)

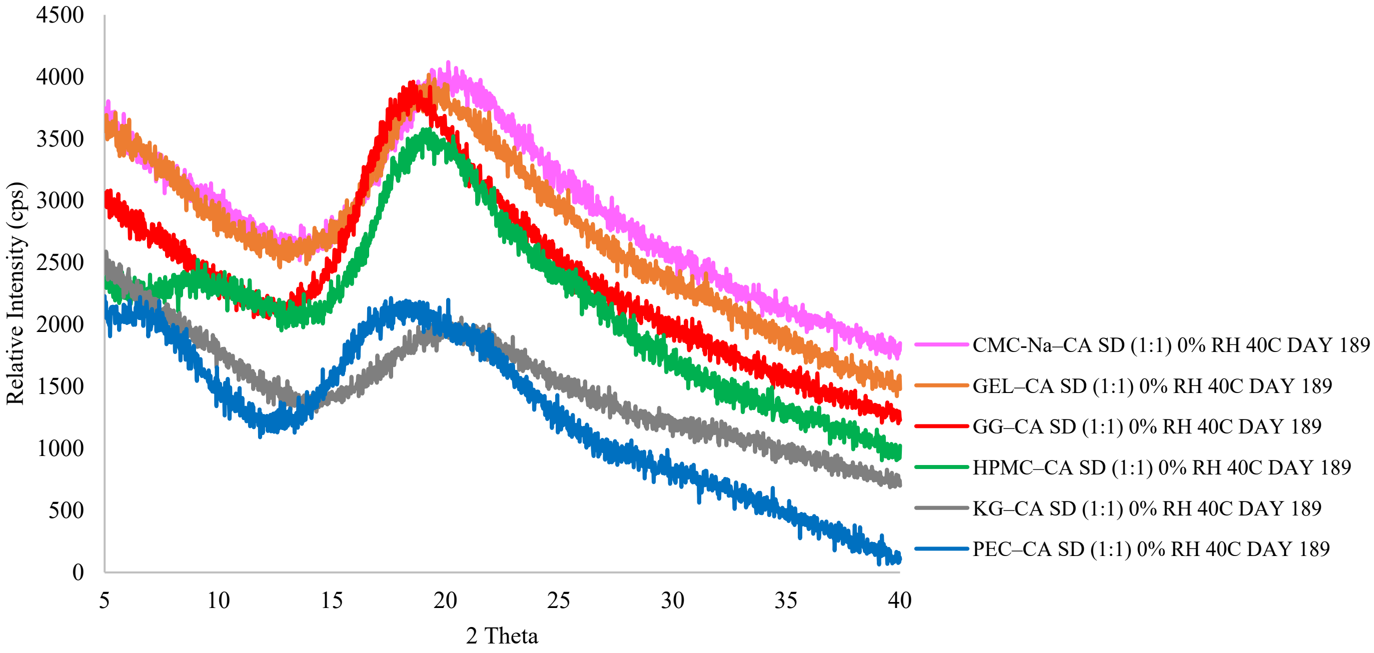
4500
4000
3500
3000
2500
2000
1500
1000
500
0
Relative Intensity (cps)
5
10
15
20
25
30
35
40
2 Theta
CMC-Na–CA SD (1:1) 0% RH 40C DAY 189
GEL–CA SD (1:1) 0% RH 40C DAY 189
GG–CA SD (1:1) 0% RH 40C DAY 189
HPMC–CA SD (1:1) 0% RH 40C DAY 189
KG–CA SD (1:1) 0% RH 40C DAY 189
PEC–CA SD (1:1) 0% RH 40C DAY 189

c)

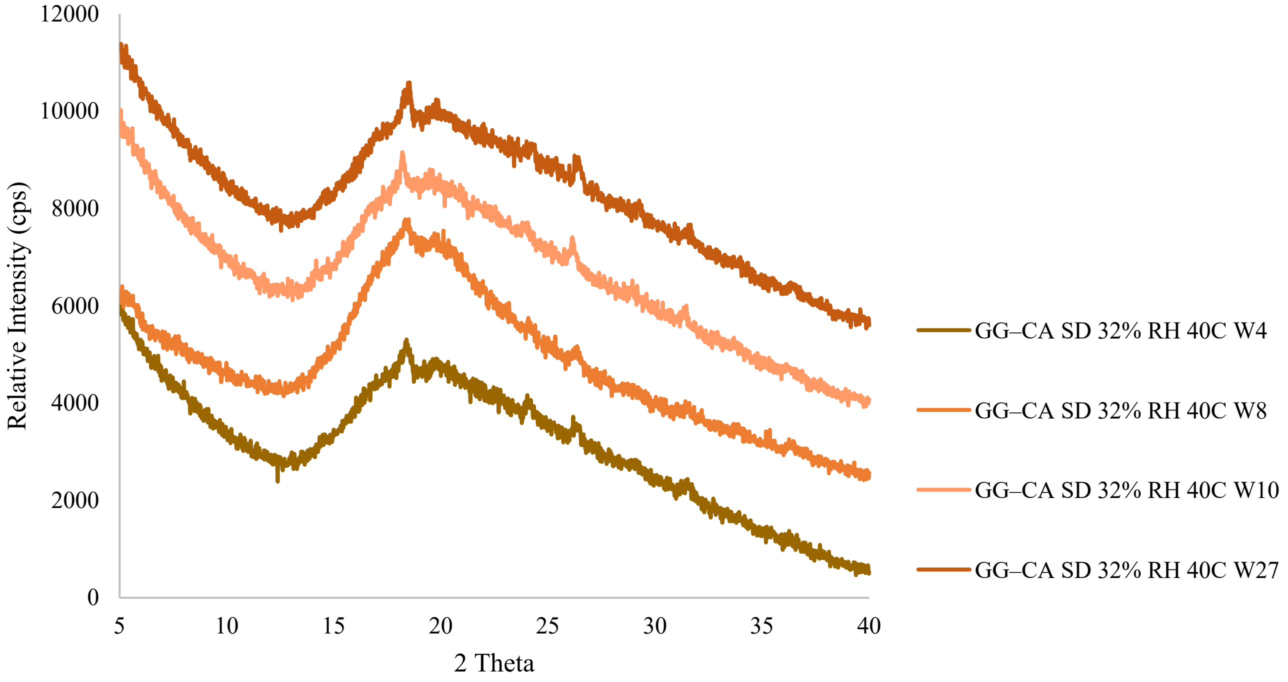

Figure S2: PXRD patterns of: a) 1:1 CA – polymer solid dispersions at 0% RH and 25 °C on day 189, b) 1:1 CA – polymer solid dispersions at 0% RH and 40 °C on day 189, c) 1:1 CA – GG solid dispersions.

a)

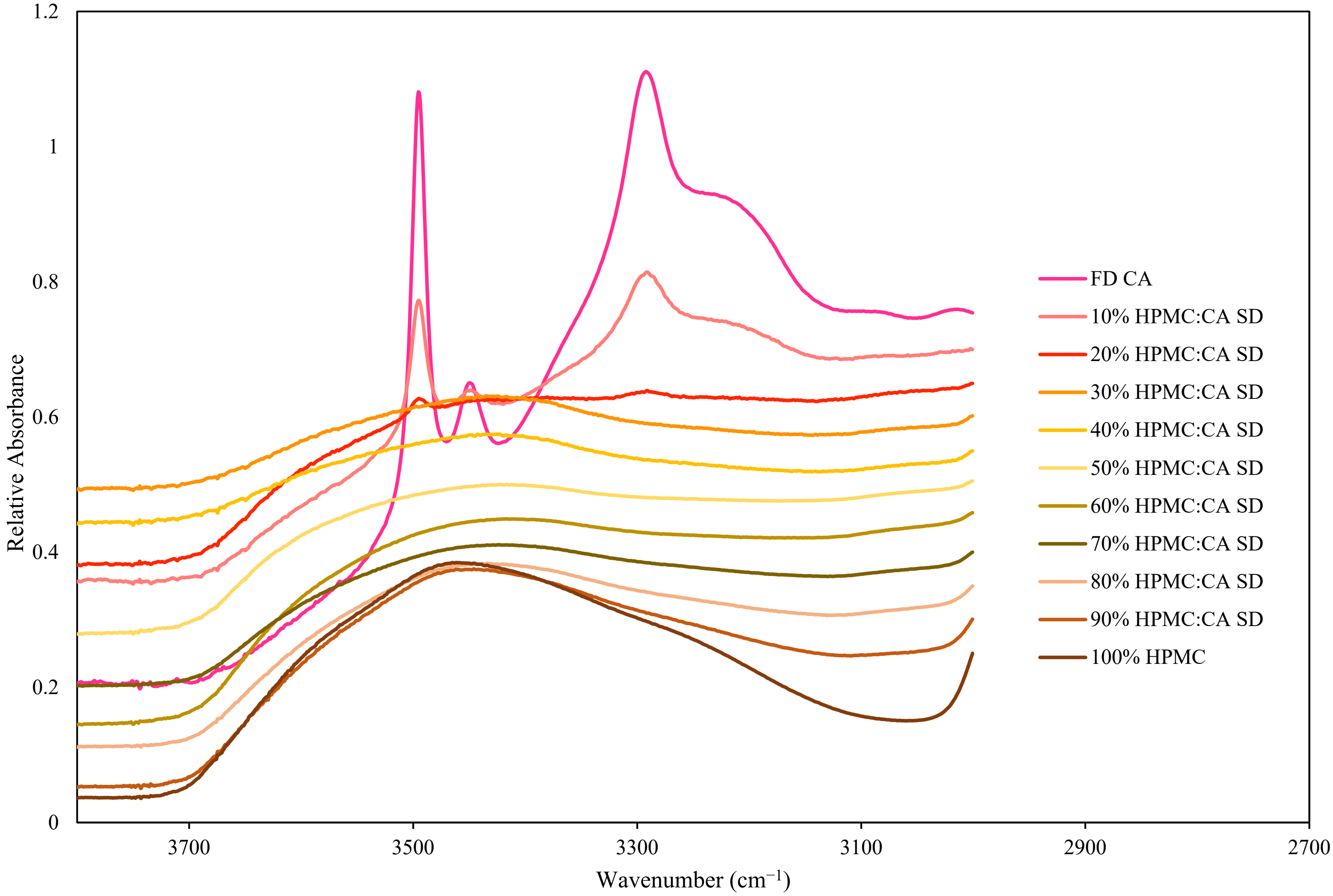

1.2
1
0.8
0.6
0.4
0.2
0
Relative Absorbance
3700
3500
3300
3100
2900
2700
Wavenumber (cm⁻¹)
FD CA
10% HPMC:CA SD
20% HPMC:CA SD
30% HPMC:CA SD
40% HPMC:CA SD
50% HPMC:CA SD
60% HPMC:CA SD
70% HPMC:CA SD
80% HPMC:CA SD
90% HPMC:CA SD
100% HPMC

b)

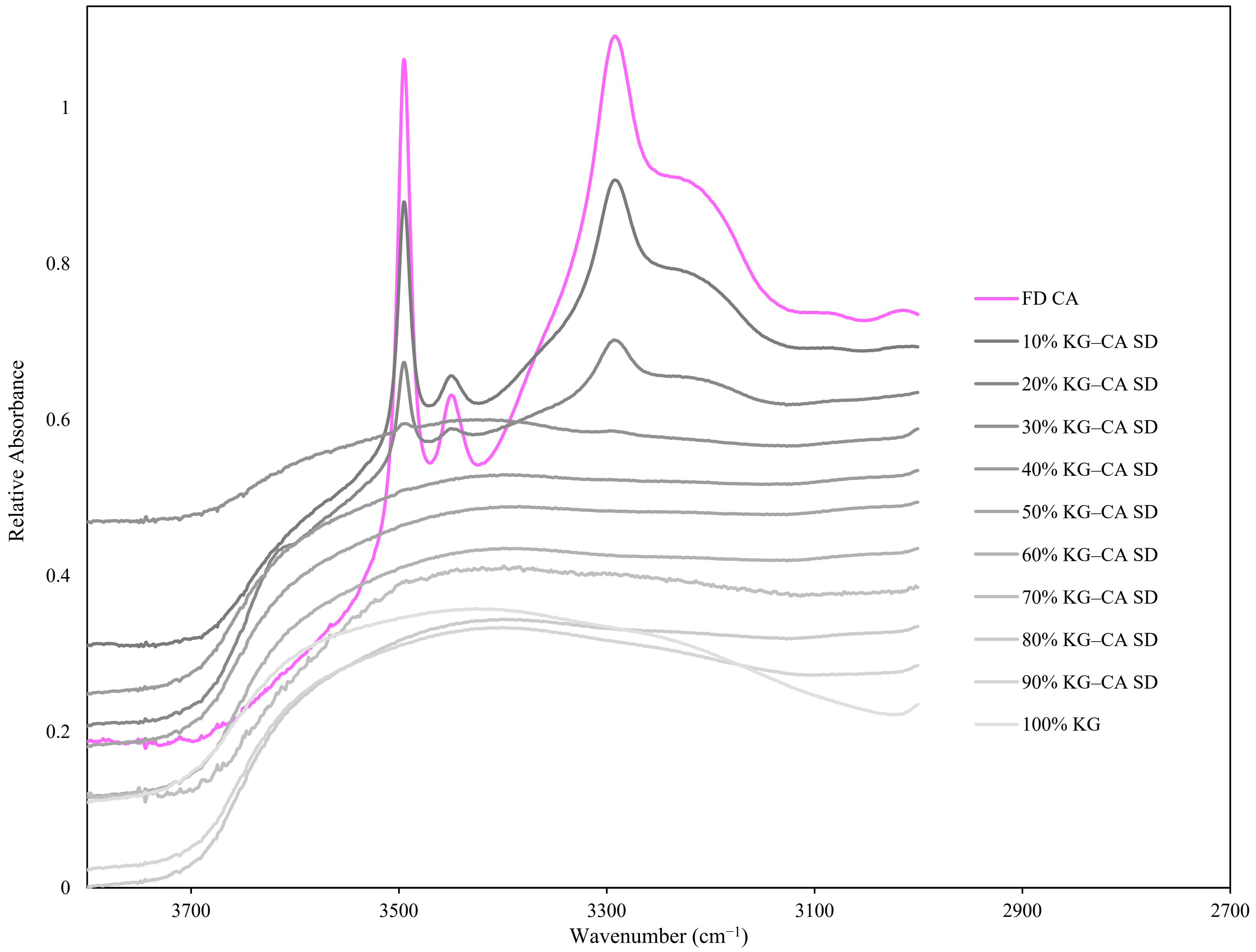

Relative Absorbance
Wavenumber (cm⁻¹)
0
0.2
0.4
0.6
0.8
1
3700
3500
3300
3100
2900
2700
FD CA
10% KG–CA SD
20% KG–CA SD
30% KG–CA SD
40% KG–CA SD
50% KG–CA SD
60% KG–CA SD
70% KG–CA SD
80% KG–CA SD
90% KG–CA SD
100% KG

c)

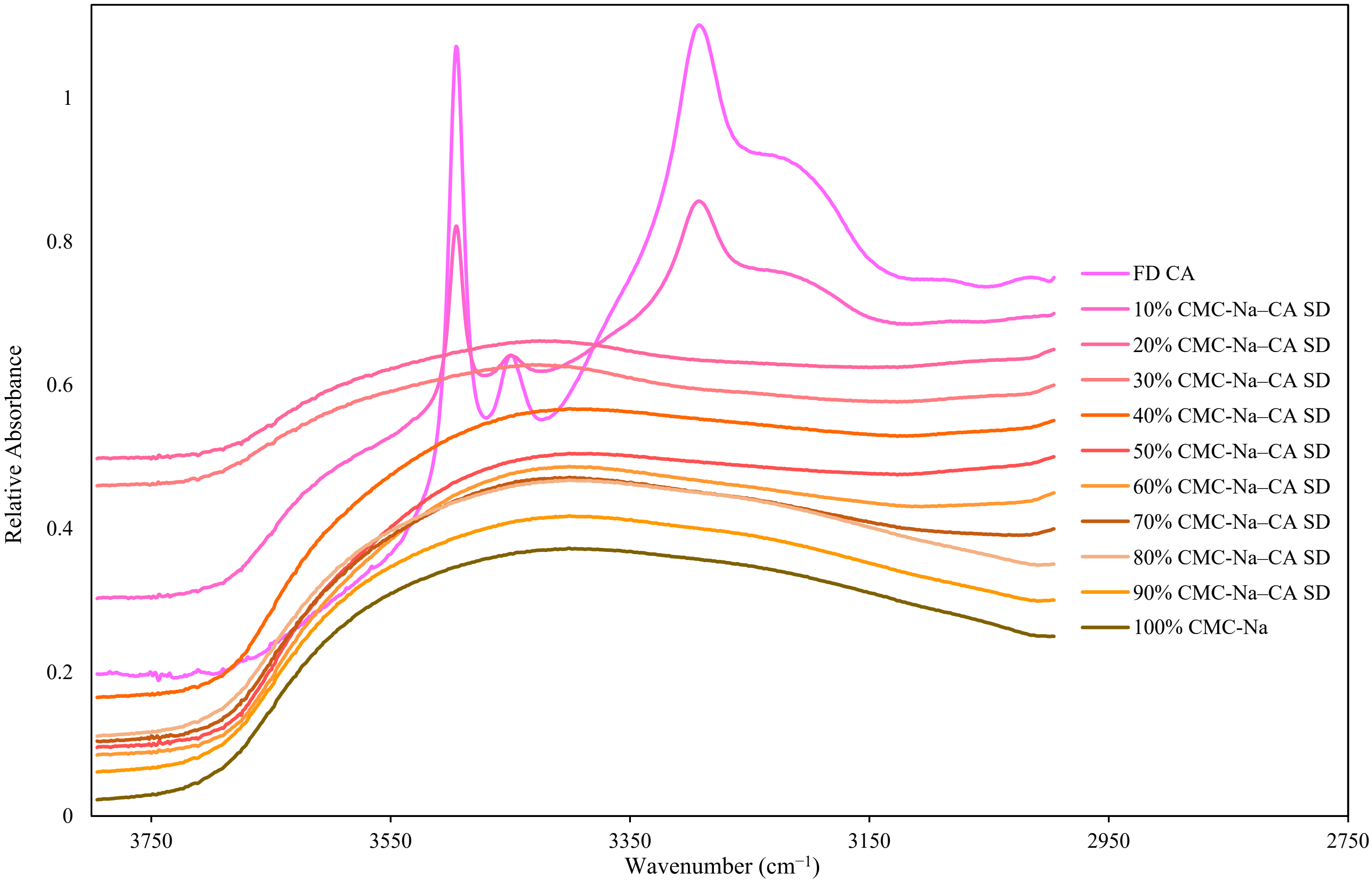

Figure S3: Mid infrared spectra of: a) CA – HMPC solid dispersions (SD), b) CA – KG solid dispersions (SD), c) CA – CMC-Na solid dispersions (SD), made with various ratios (NH/OH region is shown).

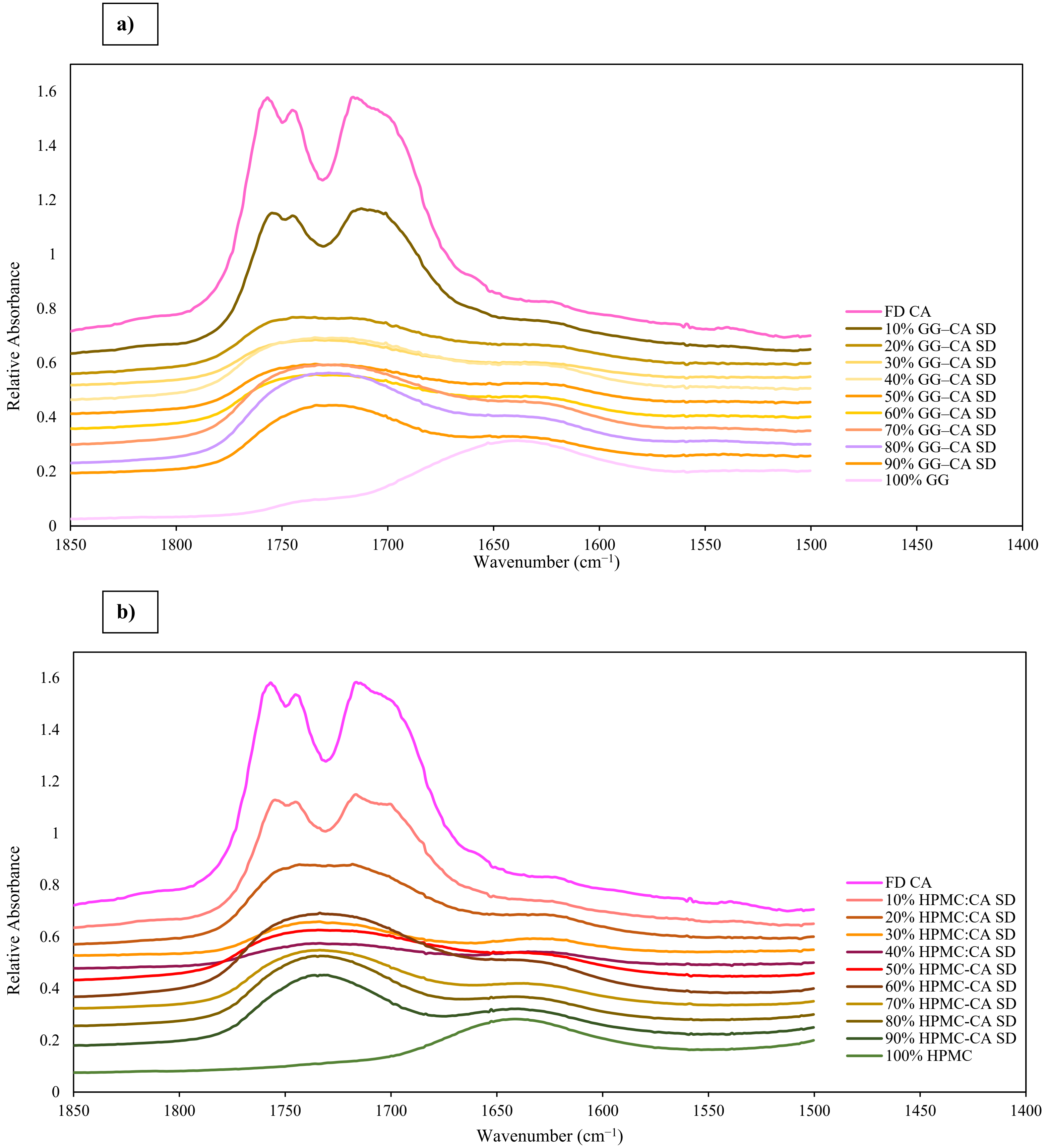
a)
Relative Absorbance
Wavenumber (cm⁻¹)
FD CA
10% GG–CA SD
20% GG–CA SD
30% GG–CA SD
40% GG–CA SD
50% GG–CA SD
60% GG–CA SD
70% GG–CA SD
80% GG–CA SD
90% GG–CA SD
100% GG
b)
Relative Absorbance
Wavenumber (cm⁻¹)
FD CA
10% HPMC:CA SD
20% HPMC:CA SD
30% HPMC:CA SD
40% HPMC:CA SD
50% HPMC-CA SD
60% HPMC-CA SD
70% HPMC-CA SD
80% HPMC-CA SD
90% HPMC-CA SD
100% HPMC

c)

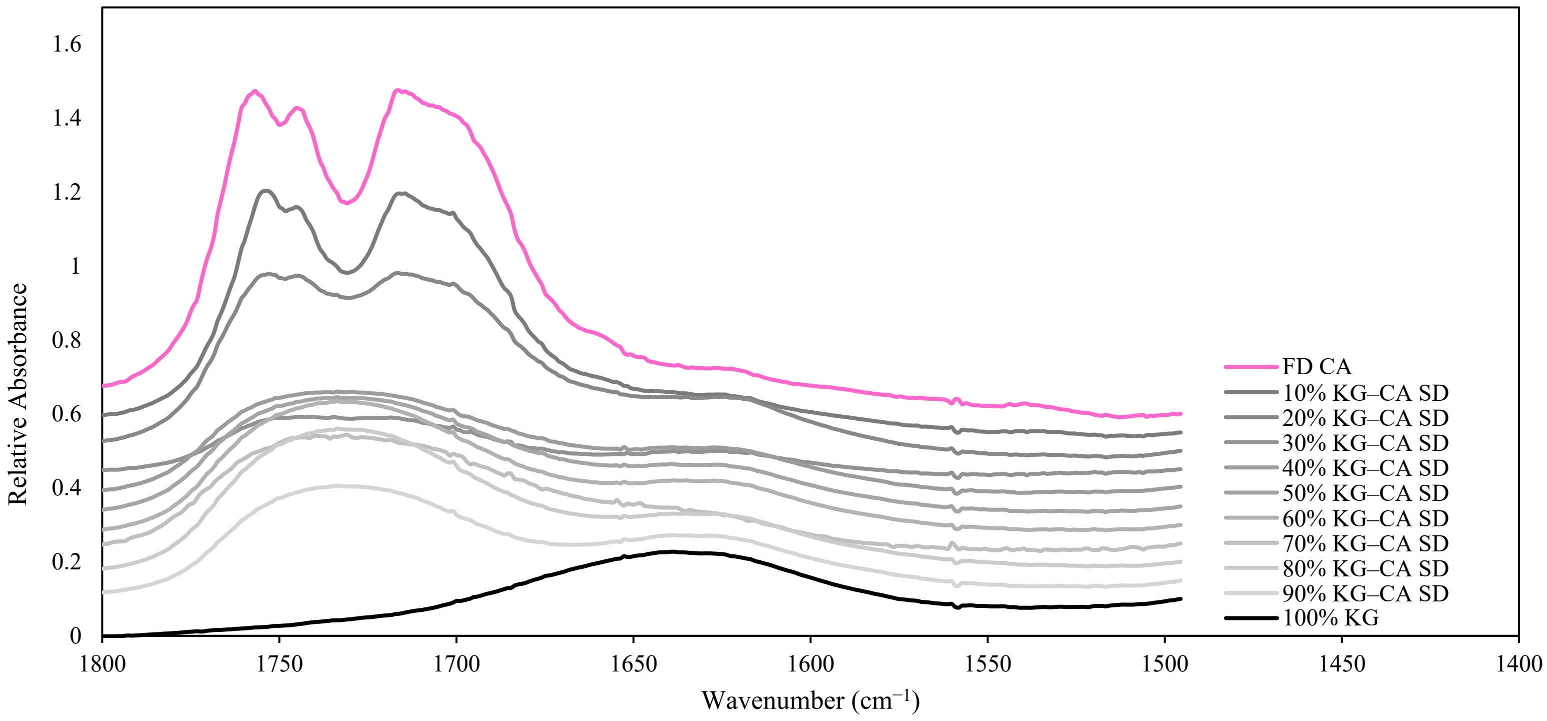

d)

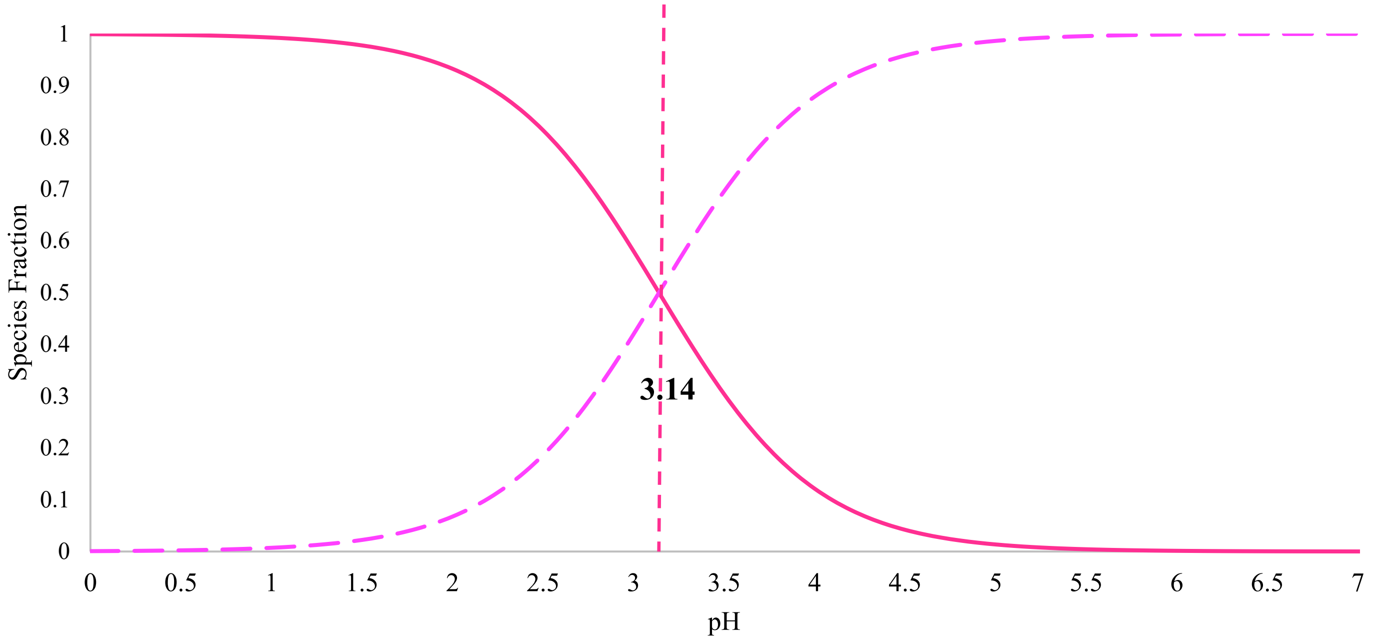

Figure S4: Mid infrared spectra of: a) CA – GG solid dispersion (SD), b) CA – HPMC solid dispersion (SD), c) CA – KG solid dispersion (SD), made with various ratios, (carbonyl region is shown), d) Speciation plot of CA.

a)

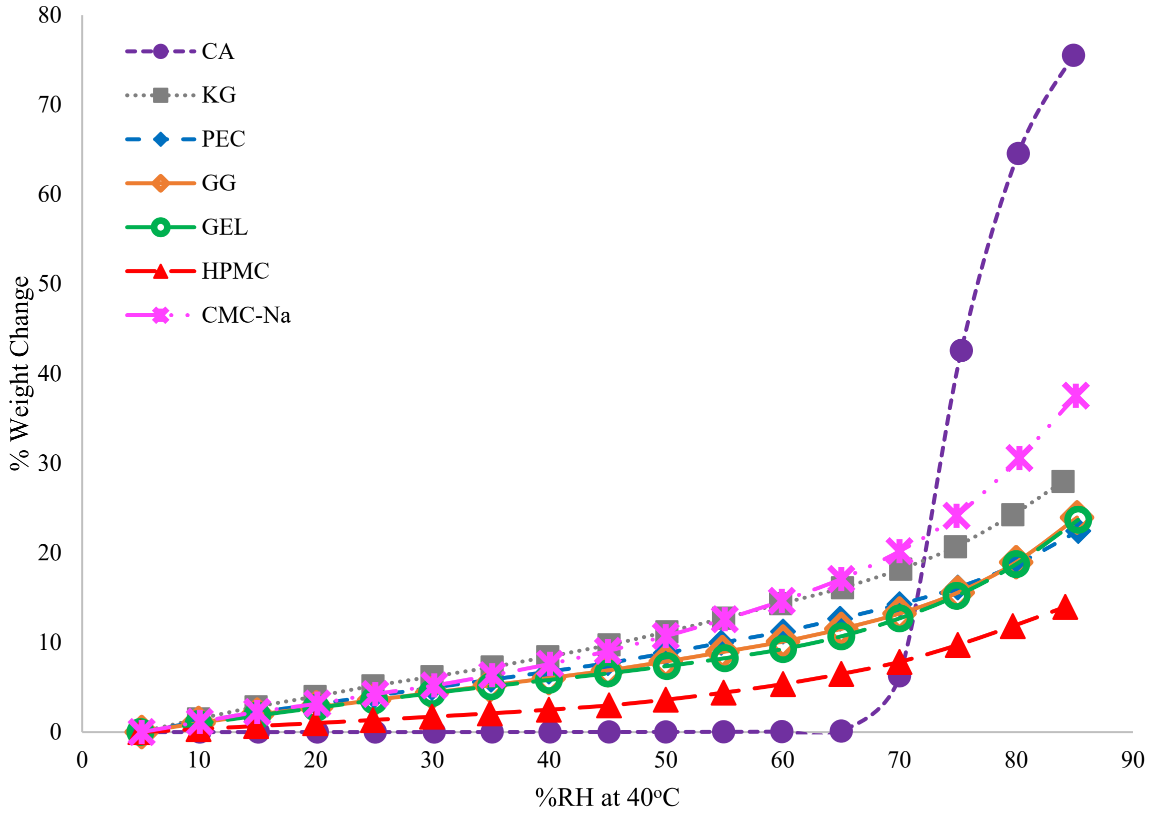
80
70
60
50
40
30
20
10
0
% Weight Change
0
10
20
30
40
50
60
70
80
90
%RH at 40°C
CA
KG
PEC
GG
GEL
HPMC
CMC-Na

b)

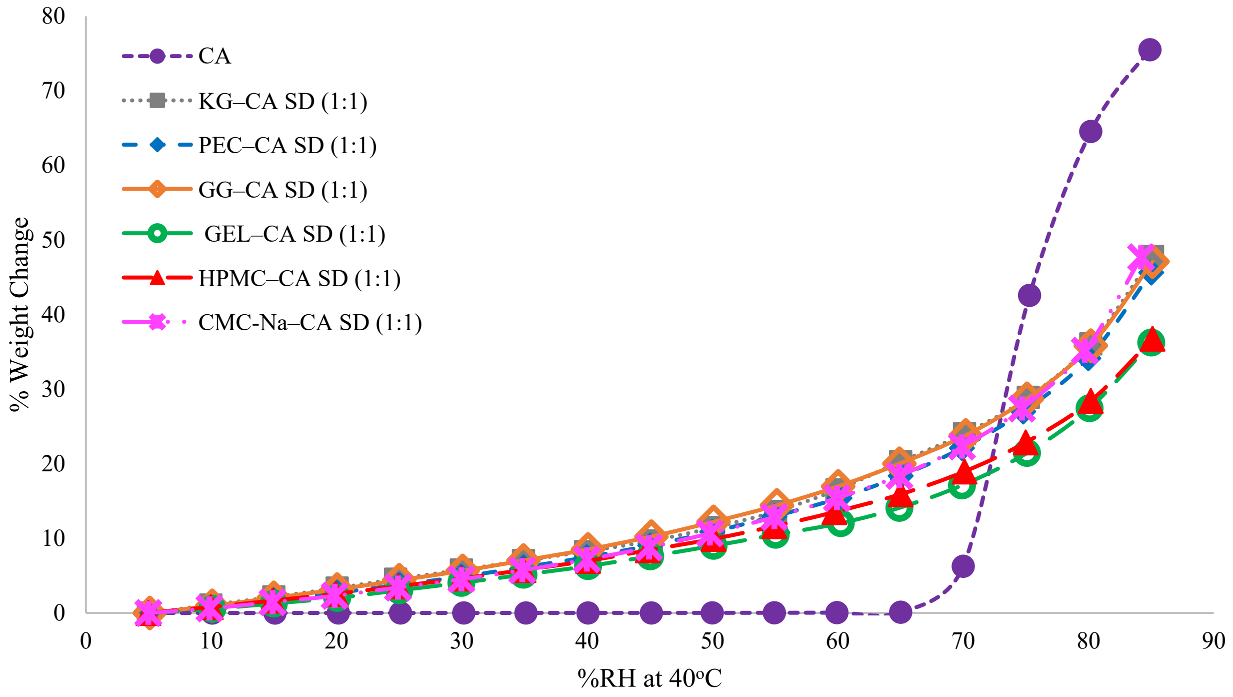

c)

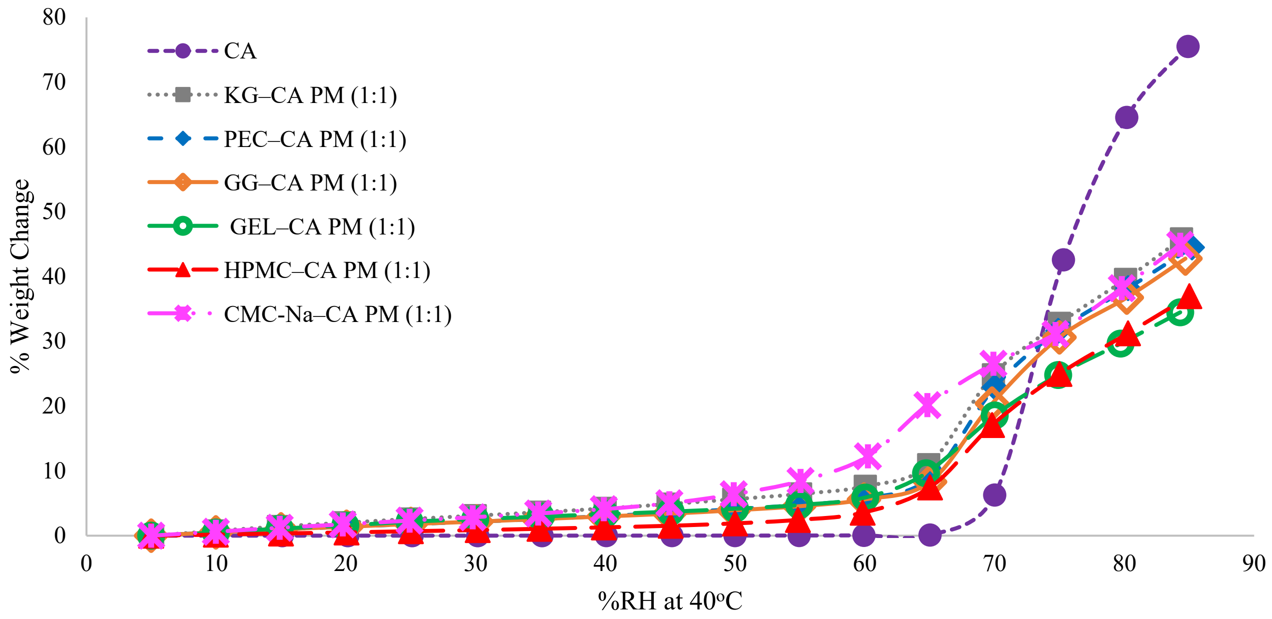

Figure S5: Moisture sorption profiles of samples at 40 °C: **(a)** CA and polymers, **(b)** 1:1 CA – polymer physical mixtures (PMs), **(c)** 1:1 CA – polymer solid dispersions (SDs).

a)

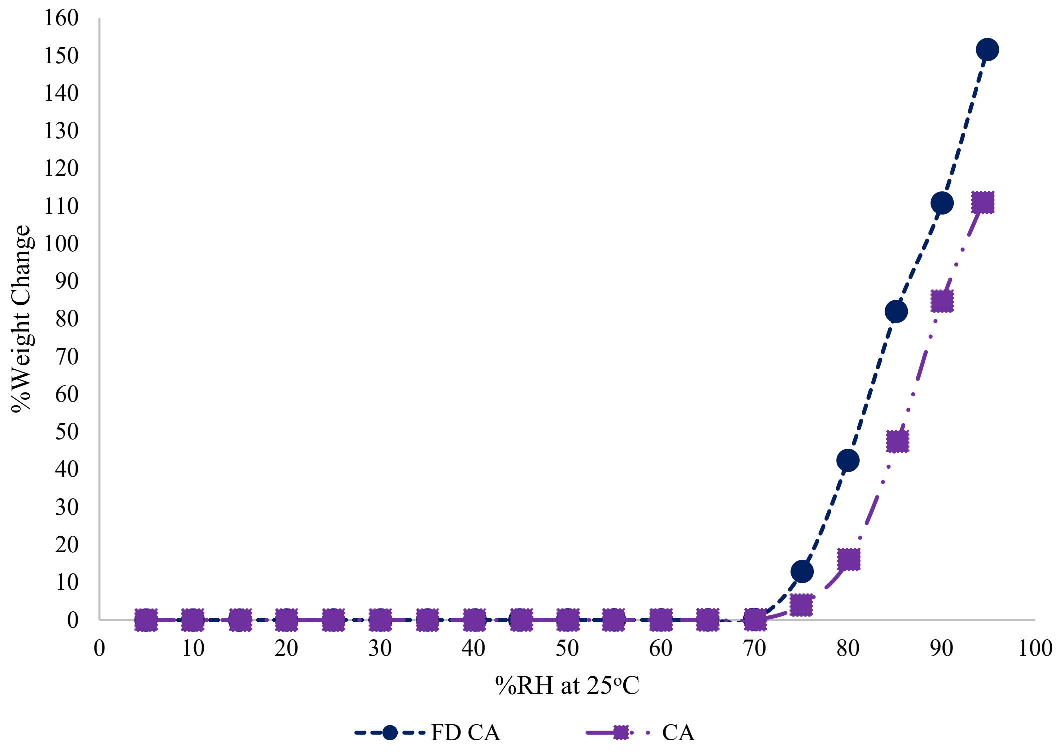
%Weight Change
%RH at 25°C
FD CA
CA

b)

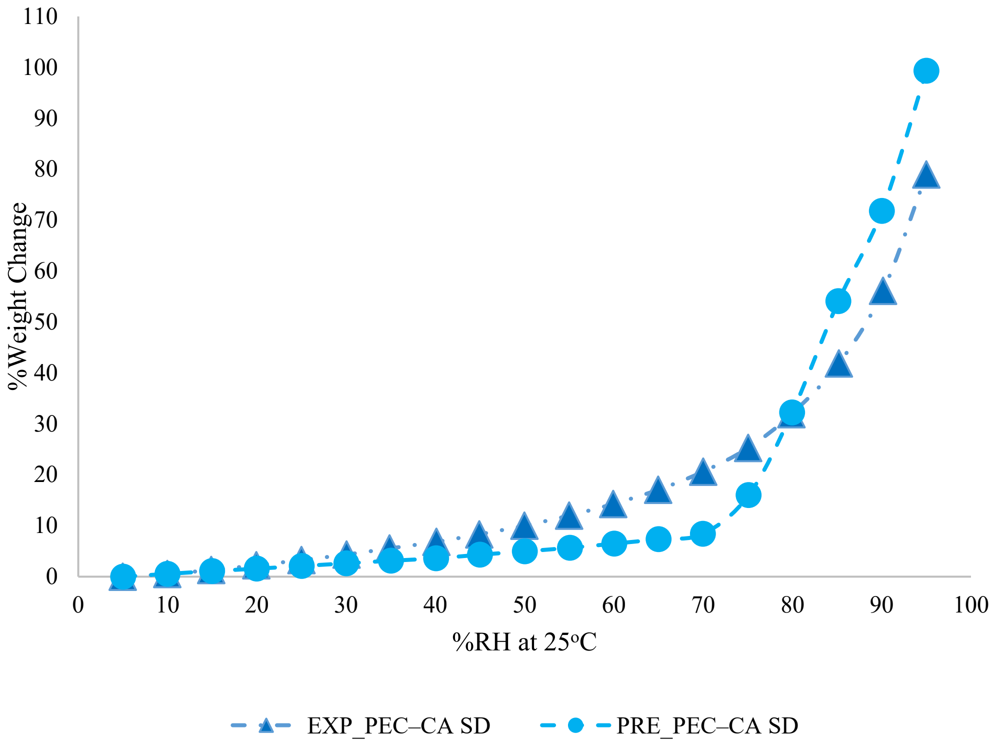
%Weight Change
%RH at 25°C
EXP_PEC–CA SD
PRE_PEC–CA SD

c)

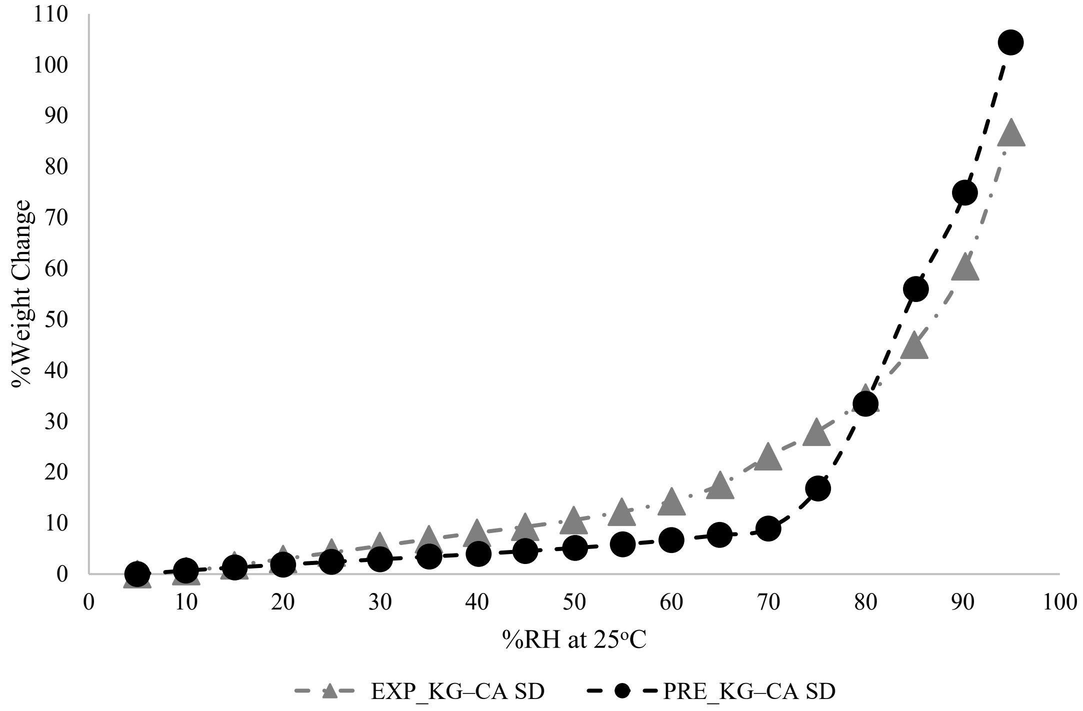
110
100
90
80
70
60
50
40
30
20
10
0
%Weight Change
0
10
20
30
40
50
60
70
80
90
100
%RH at 25°C
EXP_KG–CA SD
PRE_KG–CA SD

d)

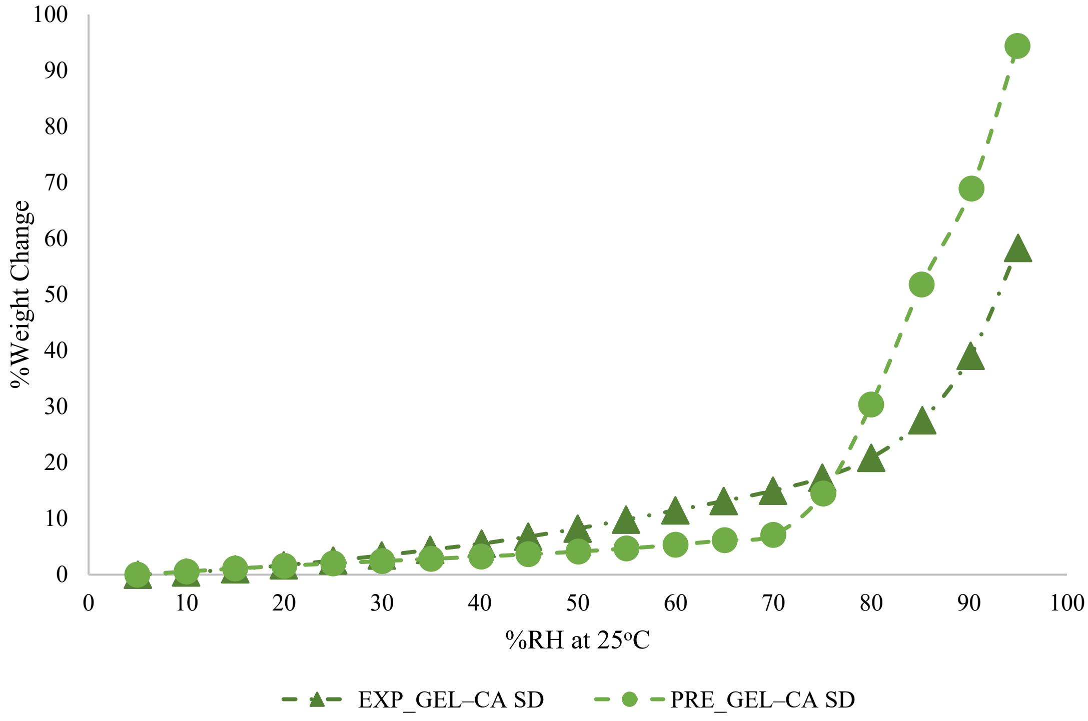
100
90
80
70
60
50
40
30
20
10
0
%Weight Change
0
10
20
30
40
50
60
70
80
90
100
%RH at 25°C
EXP_GEL–CA SD
PRE_GEL–CA SD

e)

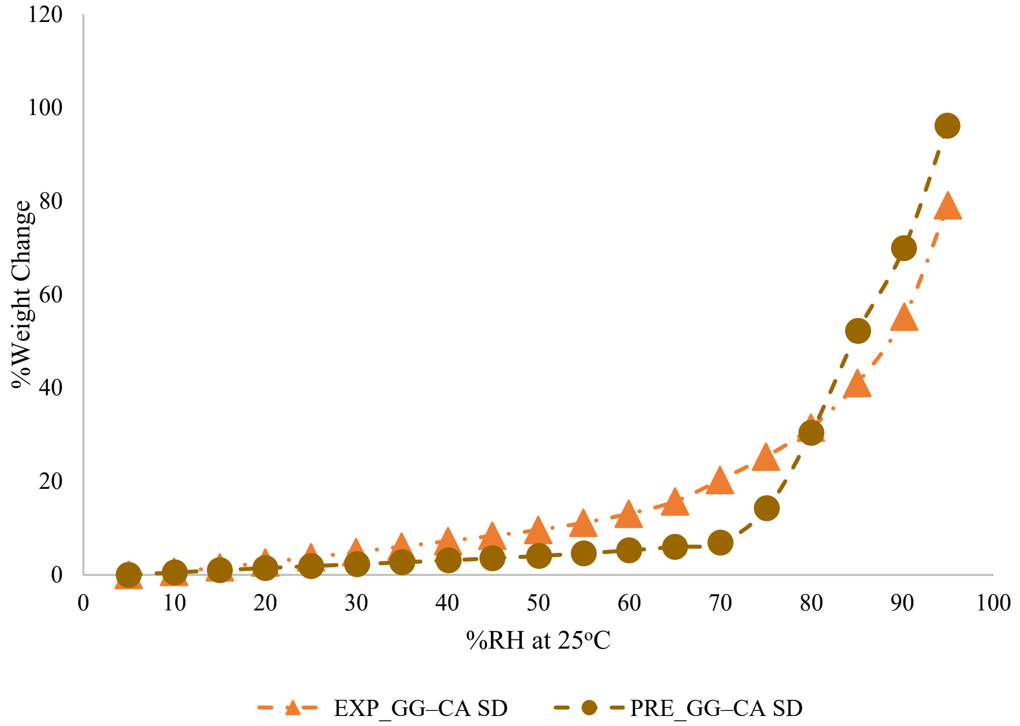
%Weight Change
%RH at 25°C
0
20
40
60
80
100
120
0
10
20
30
40
50
60
70
80
90
100
EXP_GG–CA SD
PRE_GG–CA SD

f)

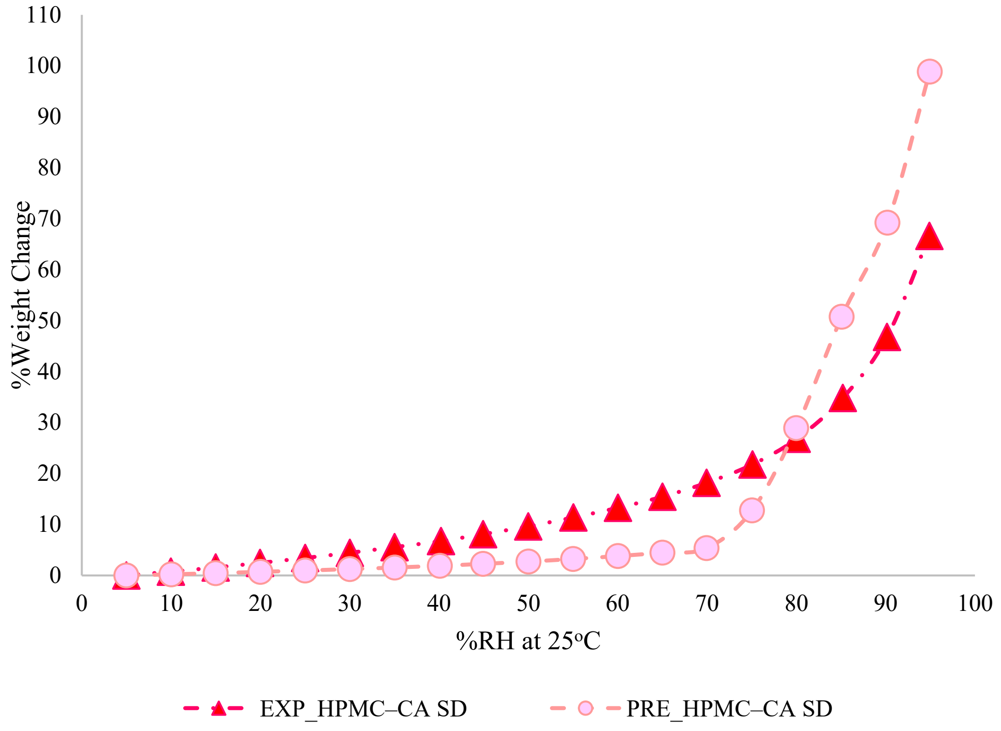
%Weight Change
%RH at 25°C
0
10
20
30
40
50
60
70
80
90
100
110
0
10
20
30
40
50
60
70
80
90
100
EXP_HPMC–CA SD
PRE_HPMC–CA SD

g)

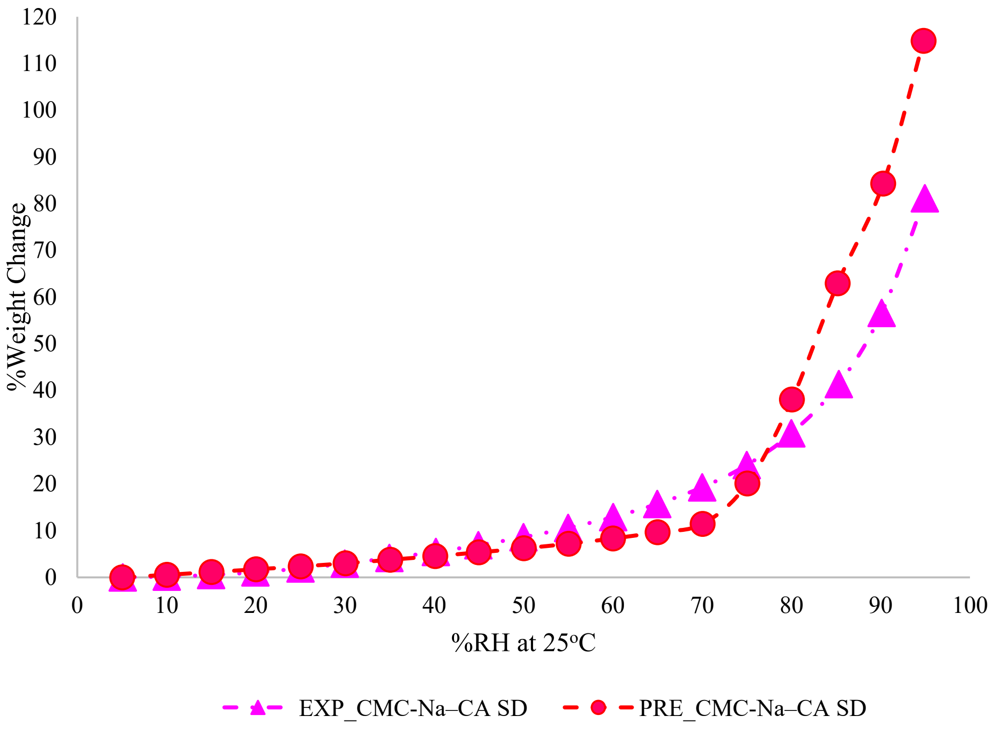

Figure S6: a) Moisture sorption profiles of samples CA and FD CA at 25 °C. Experimental (EXP) moisture sorption profile of 1:1 CA – polymer solid dispersions (SD) vs. predicted (PRE) moisture sorption profile, b) CA – PEC solid dispersions (SD) c) CA – KG solid dispersions (SD), d) CA – GEL solid dispersions (SD), e) CA – GG solid dispersions (SD), f) CA – HPMC solid dispersions (SD), g) CA – CMC-Na solid dispersions (SD).

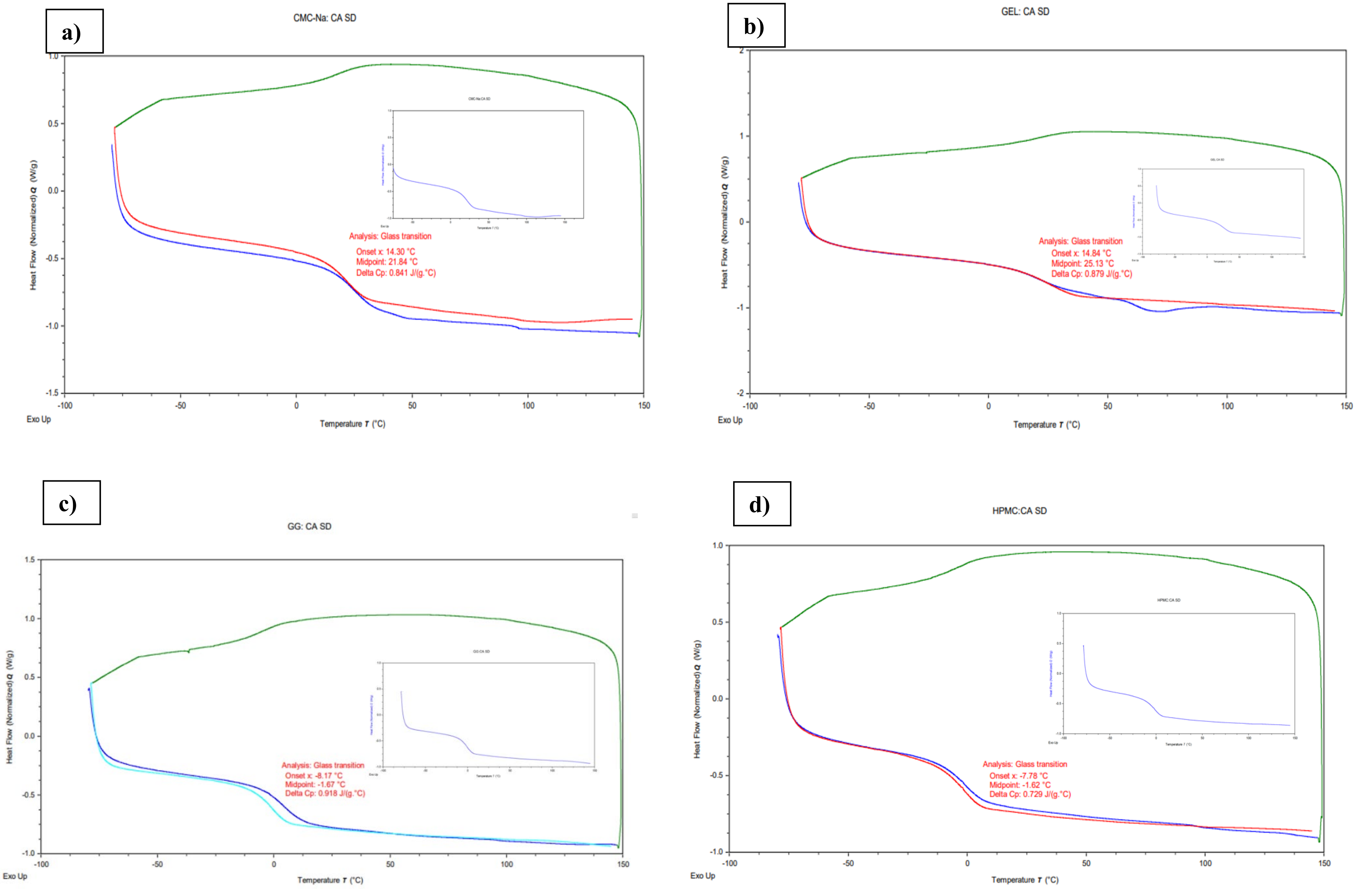
a)
CMC-Na: CA SD
Analysis: Glass transition
Onset x: 14.30 °C
Midpoint: 21.84 °C
Delta Cp: 0.841 J/(g.°C)
Heat Flow (Normalized) Q (W/g)
Temperature T (°C)
Exo Up
b)
GEL: CA SD
Analysis: Glass transition
Onset x: 14.84 °C
Midpoint: 25.13 °C
Delta Cp: 0.879 J/(g.°C)
Heat Flow (Normalized) Q (W/g)
Temperature T (°C)
Exo Up
c)
GG: CA SD
Analysis: Glass transition
Onset x: -8.17 °C
Midpoint: -1.67 °C
Delta Cp: 0.918 J/(g.°C)
Heat Flow (Normalized) Q (W/g)
Temperature T (°C)
Exo Up
d)
HPMC:CA SD
Analysis: Glass transition
Onset x: -7.78 °C
Midpoint: -1.62 °C
Delta Cp: 0.729 J/(g.°C)
Heat Flow (Normalized) Q (W/g)
Temperature T (°C)
Exo Up

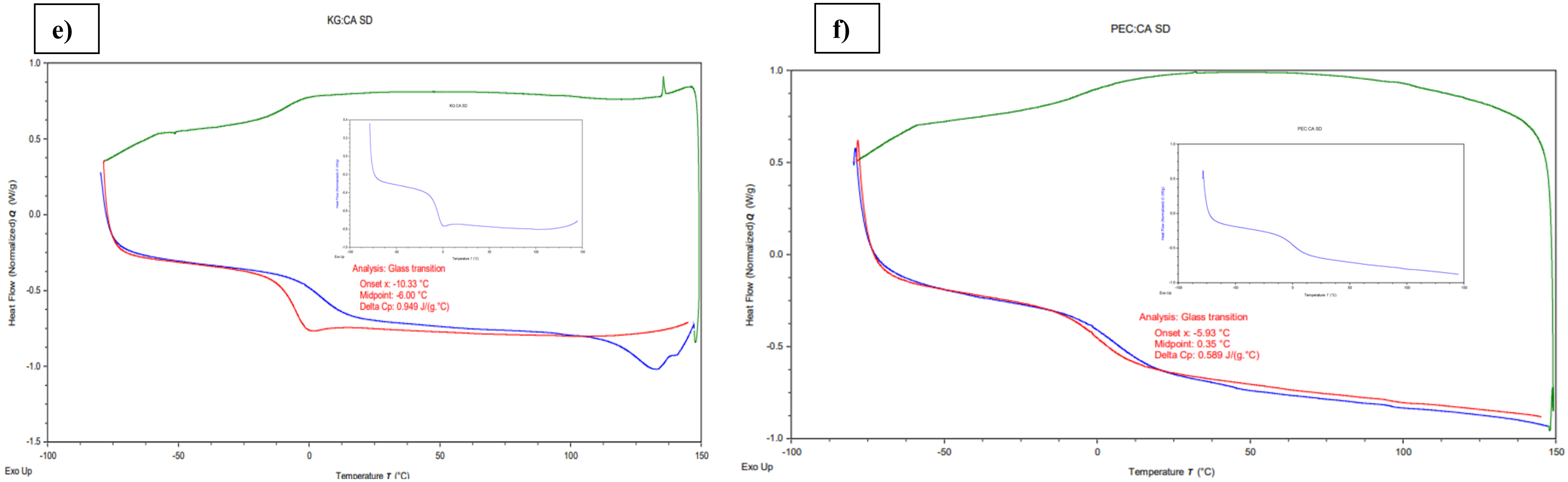

Figure S7: DSC thermograms of solid dispersions without pin hole (The separate display of second scans is shown in the full scan graphs): a) CA – CMC-Na solid dispersions (SD), b) CA – GEL solid dispersions (SD), c) CA – GG solid dispersions (SD), d) CA – HPMC solid dispersions (SD), e) CA – KG solid dispersions (SD), f) CA – PEC solid dispersions (SD).

a)

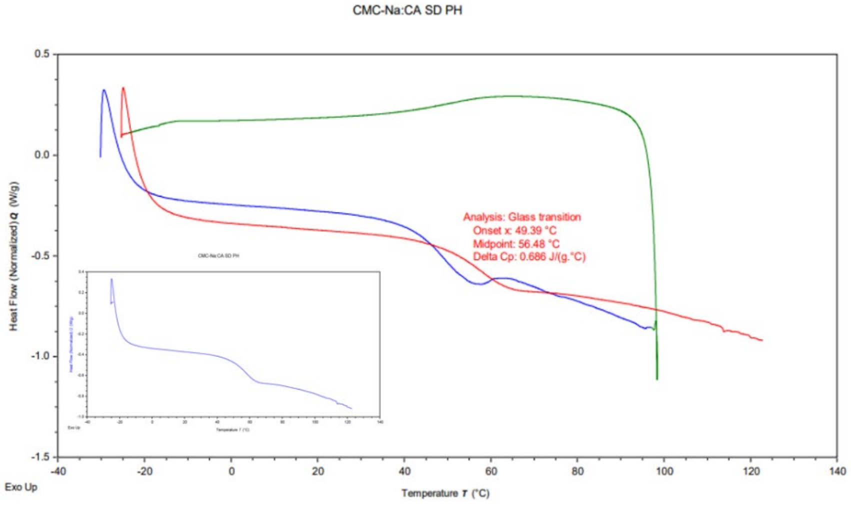
CMC-Na:CA SD PH
Heat Flow (Normalized) Q (W/g)
Analysis: Glass transition
Onset x: 49.39 °C
Midpoint: 56.48 °C
Delta Cp: 0.686 J/(g.°C)
Exo Up
Temperature T (°C)

b)

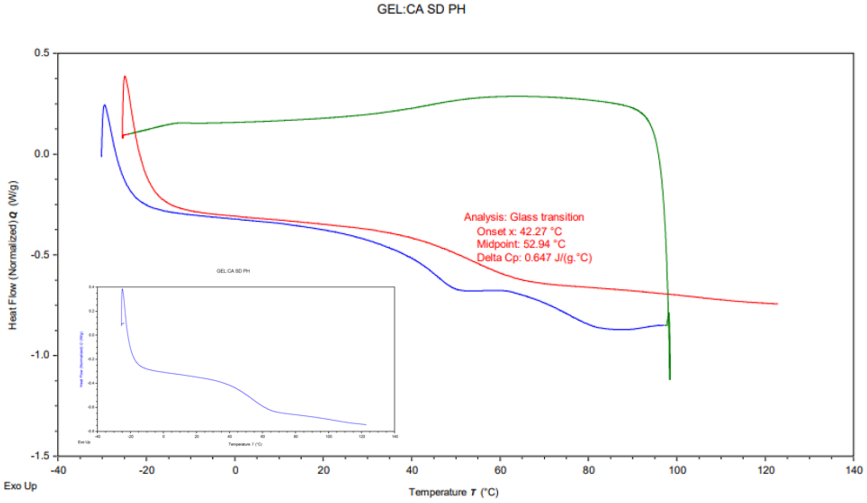
GEL:CA SD PH
Heat Flow (Normalized) Q (W/g)
Analysis: Glass transition
Onset x: 42.27 °C
Midpoint: 52.94 °C
Delta Cp: 0.647 J/(g.°C)
Exo Up
Temperature T (°C)

c)

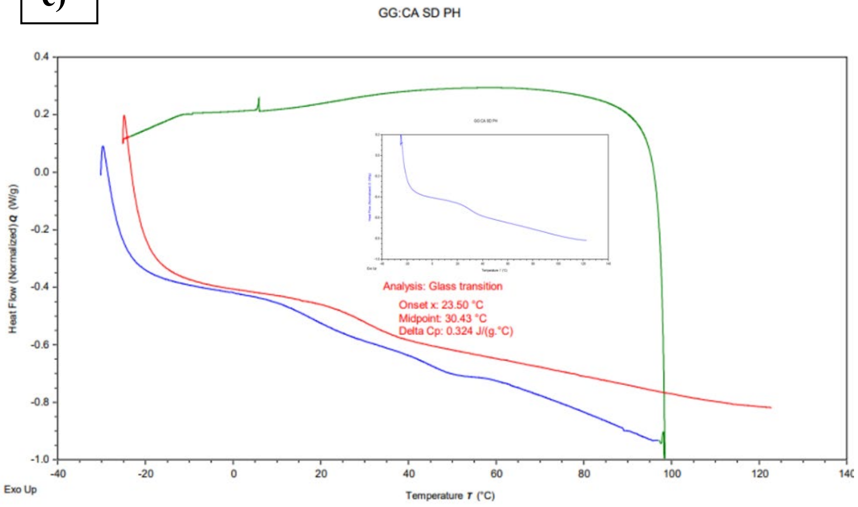
GG:CA SD PH
Heat Flow (Normalized) Q (W/g)
Analysis: Glass transition
Onset x: 23.50 °C
Midpoint: 30.43 °C
Delta Cp: 0.324 J/(g.°C)
Exo Up
Temperature T (°C)

d)

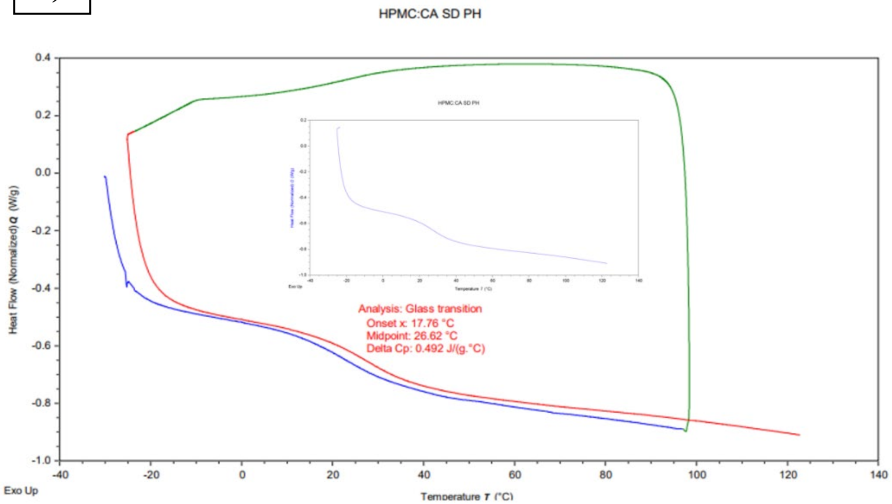
HPMC:CA SD PH
Heat Flow (Normalized) Q (W/g)
Analysis: Glass transition
Onset x: 17.76 °C
Midpoint: 26.62 °C
Delta Cp: 0.492 J/(g.°C)
Exo Up
Temperature T (°C)

e)

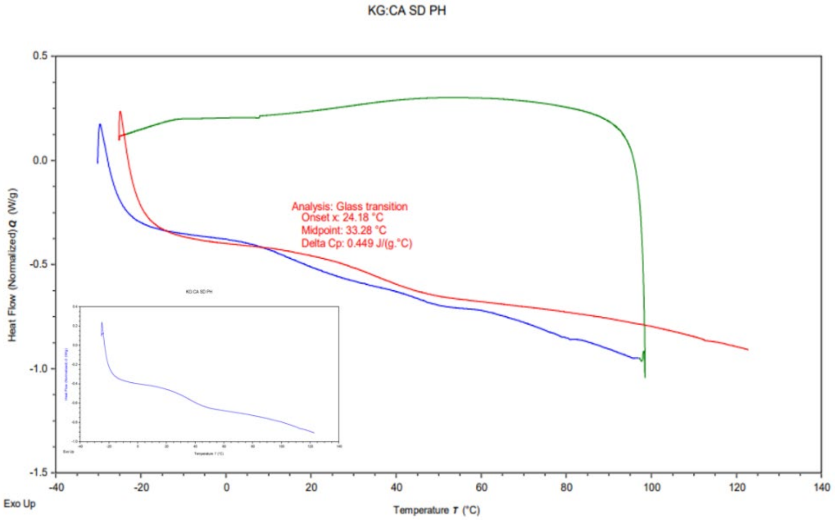

f)

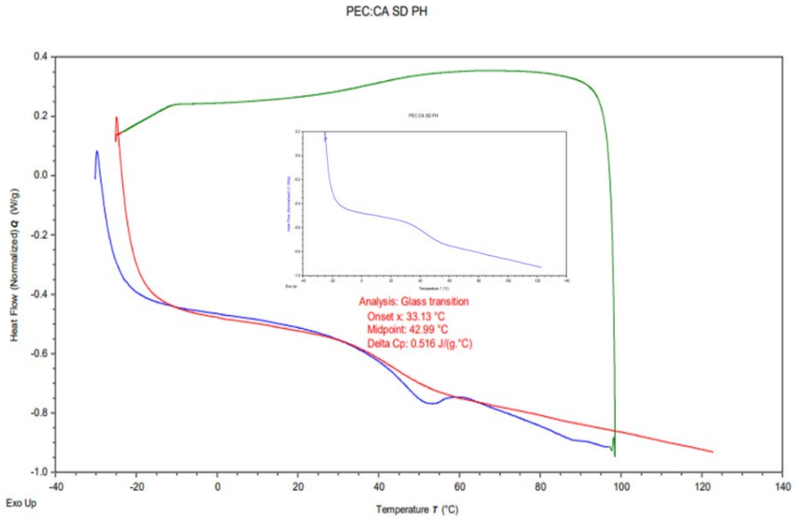

Figure S8: DSC thermograms of solid dispersions with pin hole (The separate display of second scans is shown in the full scan graphs): a) CA – CMC-Na solid dispersions (SD), b) CA – GEL solid dispersions (SD), c) CA – GG solid dispersions (SD), d) CA – HPMC solid dispersions (SD), e) CA – KG solid dispersions (SD), f) CA – PEC solid dispersions (SD).
